# Multichannel, biomimetic scaffolds improve functional connectivity following traumatic long-gap peripheral nerve injury

**DOI:** 10.1038/s41467-026-77537-6

**Published:** 2026-09-21

**Authors:** Jacob Koffler, Eleni Sinopoulou, Isac Lazarovits, Deborah M. W. Alexander, Alec Salcido, Kendell Pawelec, Reid Abrams, Jeff Sakamoto, Mark H. Tuszynski

**Affiliations:** 1 Department of Neuroscience, University of California San Diego, La Jolla, CA USA; 2 Veterans Affairs Medical Center, San Diego, CA USA; 3 Department of Radiology & Institute for Quantitative Health Science & Engineering, Michigan State University, East Lansing, MI USA; 4 Department of Orthopedics, University of California San Diego, La Jolla, CA USA; 5 Materials Department, University of California Santa Barbara, Santa Barbara, CA USA

**Keywords:** Regeneration and repair in the nervous system, Biomedical engineering, Peripheral nervous system, Implants

## Abstract

The gold standard for repair of peripheral nerve injuries is a nerve autograft. Donor nerve harvest creates a second surgical site, invariable numbness and donor site chronic neuropathic pain in 20% of cases. We developed a poly-caprolactone (PCL) multichannel, biomimetic scaffold with surface features promoting cell and vascular attachment and rapid, linear axonal regeneration. The cross-sectional area available for axonal regeneration exceeds that of autografts. When compared to nerve autografts and FDA-approved nerve conduits in 1cm-long rat and 3cm-long rabbit sciatic gap injury, biomimetic scaffolds were equivalent or superior to nerve autografts on measures of axonal regeneration, re-connection between gastrocnemius muscle and spinal cord motor neurons, and evoked motor responses. Scaffolds were superior in all measures to open tube conduits. Biomimetic multichannel scaffolds have potential to replace the need for autografting for peripheral nerve gap repair and are now undergoing multicenter clinical trials.

## Abstract (web summary)

Peripheral nerve injuries are typically repaired using nerve autografts despite donor-site morbidity. Here, the authors show that a biomimetic multichannel scaffold that organizes axonal regeneration, matches or surpasses autografts performance in preclinical models.

## Introduction

Peripheral nerve injuries occur in 3% of trauma cases and 30% of all combat injuries, leading to an estimated 563,000 repair surgeries each year in the U.S^1–3^. The total annual cost is estimated to reach $75 billion, factoring in direct medical expenses as well as productivity losses from missed workdays and early retirement, long-term disability and income replacement, and the costs of caregiver support for individuals left with lasting impairments^1–3^. In short nerve gap injuries under 5 mm in length, neurorrhaphy, or direct suture repair, is the most common surgical intervention^4^. However, injuries that cause defects longer than 5 mm require an implant to bridge the injury site, as the tension caused by neurorrhaphy over these distances results in poor surgical outcomes^5^.

The current gold standard for peripheral nerve gap repair is the nerve autograft, and most commonly, the sural nerve is used to bridge the injury site^6^. Harvesting nerve autografts requires a second surgical procedure, causing donor site morbidity and loss of function. The sural nerve has a relatively low percentage of neural tissue cross-sectional area (~33%), limiting the area for regenerating axons to traverse as they navigate to the distal nerve^7^. Mismatch of donor nerve size with the injured nerve site often leads to multiple autograft segments needed for repair^8^. In addition, the consequences of this procedure are loss of sensory function of the donor nerve with resulting permanent numbness in all patients; chronic pain occurs in up to 20% of cases^9^. Thus, an alternative of equal efficacy to autografts is needed and has been identified by surgeons as a major unmet medical need.

These challenges have led to the development of nerve conduits to facilitate peripheral nerve repair in transection injuries. Current FDA-approved devices are designed as open tubes, using several materials including polyvinyl alcohol hydrogels, type I collagen (Neuragen Nerve Guide from Integra LifeSciences), polyglycolic acid, and poly _D,L_ lactide-*co*-ε-caprolactone^10–18^. While single-lumen nerve conduits offer their full cross-sectional area for axon regeneration, they lack nerve fascicle-like structures to guide regenerating axons across the injury site. This causes axon misdirection, which limits regenerative potential and results in a lack of functional recovery^19–21^ Newer versions of those products are open tubes filled with amorphous collagen, such as NeuraGen 3D^13^ or Nerbridge^17^; however, these still lack physical guidance, and preclinical studies in rat sciatic nerve injury models demonstrate that they remain inferior to nerve autografts^12,16^. No clinical data with the latter devices are available at this time.

A newer biologic alternative is the human cadaveric nerve allograft^11^, currently sold by AxoGen Inc. as “Avance”^22^. In animal studies with gaps of 14–28 mm, Avance was inferior to autografts and comparable to the Neuragen open tube, without improved functional outcomes^23^. Additional preclinical studies in a rat sciatic nerve injury model (10–14 mm long) reported that it was inferior to autografts; it supported significantly fewer regenerating axons, and inferior motor recovery measured by muscle force^24^. Results of two clinical studies regarding Avance were recently provided to the FDA to request BLA device approval. The first study was a retrospective chart review (RANGER study^25^). Due to flaws in study design (e.g., no blinding or proper control group), the FDA determined the data could not serve as evidence of allografts’ effectiveness^26^. The second study (RECON^27^) was a prospective, randomized, blinded clinical trial comparing Avance allograft to the Neuragen tube, which is a simple open tube device. Surprisingly, a comparison was not made to an autograft. The RECON study showed no difference between Avance and the open tube on the primary endpoint, the sensory outcome measure of two-point discrimination (2PD) at nerve gaps less than 15 mm, indicating a lack of efficacy at this short distance. At longer gaps of 15–25 mm, the sensory outcome of 2-point discrimination was 7.5 mm ± 2.4 mm for tubes and 6.1 mm ± 3.3 mm for Avance allografts, a 1.4 point difference (*P *< 0.05)^28^; benefits in motor outcomes were not found. Notably, interobserver variation in static 2-point discrimination is commonly 1–2 mm^29^, raising the possibility that the difference in outcome at gaps longer than 15 mm was not meaningful. Nonetheless, the FDA provided approval for treatment of *sensory* nerve injury under 25 mm in length, and accelerated approval for treatment of *sensory* nerve injury greater than 25 mm in length in mixed and motor nerve injuries; however, this approval is conditional on performing another clinical trial where Avance allograft will be compared to a nerve autograft, to be completed by 2030 with high quality and more rigorous trial design and interpretation. Overall, the clinical and preclinical data are convergent in suggesting that the benefits of Avance above an open tube are limited. Thus, there remains a critical need for devices capable of supporting nerve regeneration across gaps exceeding 5 mm as an alternative to autografts and cadaveric allografts.

We previously reported the fabrication of scaffolds containing strictly linear multichannel architecture that are able to guide regenerating axons across sites of peripheral nerve injury^30–32^. The scaffold is composed of polycaprolactone (PCL), which is a biodegradable polyester, and was chosen for its superior stiffness and strength compared to hydrogels, excellent biocompatibility, and in vivo degradation that ranges from 1 to 2 years, allowing the regenerated nerve to fully reintegrate^33^. However, the fabrication of these scaffolds was labor-intensive, requiring time-consuming individual microchannel fabrication and assembly. Moreover, in vivo testing of scaffolds was limited to short time periods and short nerve gaps of 1 cm.

Here, we report the development of a new embossing technique for scaffold manufacturing that substantially simplifies and improves the efficiency of fabrication and improves the internal architecture of scaffolds by increasing the cross-sectional open area available for axon regeneration. The process involves the transformation of flat two-dimensional sheets of porous PCL into highly ordered three-dimensional architectures using a combination of embossing to form channels, followed by spiral winding (detailed description is in the “Methods” section). We tested these scaffolds in vivo over gap lengths of 1 cm in rats and 3 cm in rabbits over extended time periods of 6 and 5 months, respectively, with assessment of anatomical and functional outcomes. We now observe an advanced level of recovery at all nerve gap lengths, and equal or superior recovery when compared to gold-standard nerve autografts at the longest gap distances. Thus, these biomimetic scaffolds meet the goal of performing equivalently to nerve autografts and are now undergoing multicenter, randomized, controlled clinical trials. Our biomimetic scaffolds have the potential to eliminate the need for nerve autografting, thereby reducing the side effects of nerve harvest and reducing operative time by eliminating the need for autograft harvest.

## Results

### Embossing creates uniform and improved PCL microchannel scaffolds

**Fig. 1:**
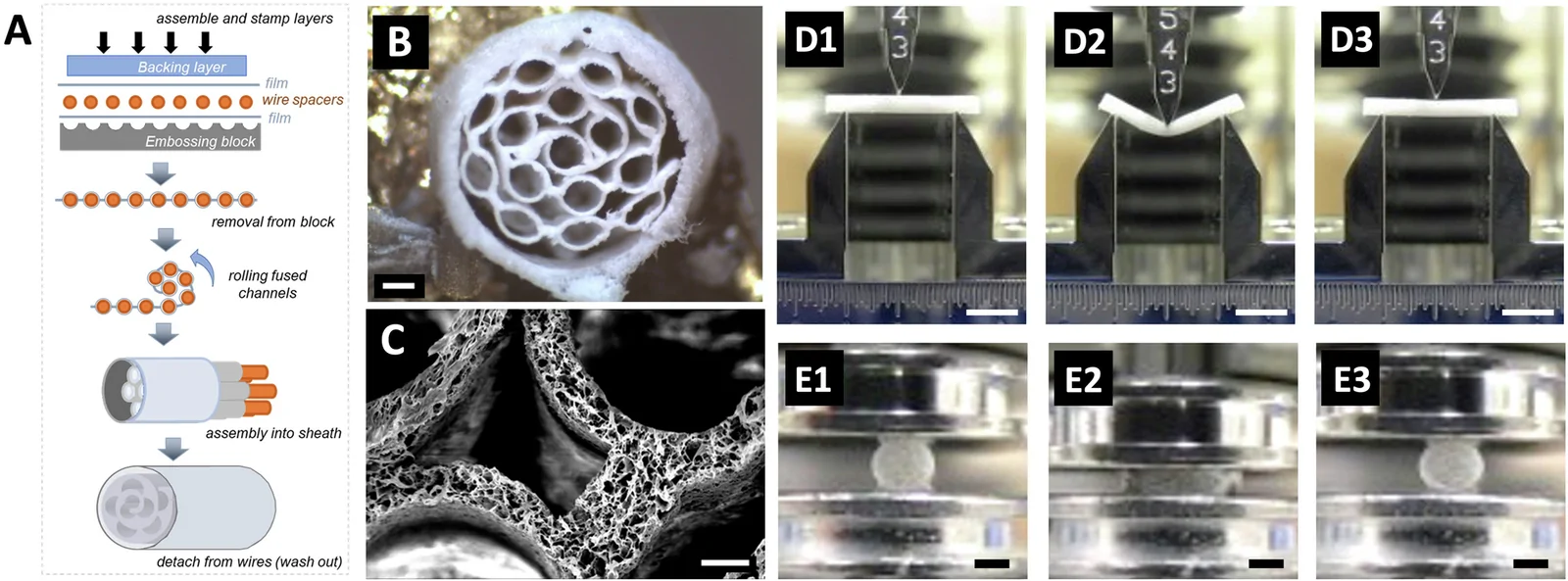
Manufacturing and characterization of embossed scaffolds. **A** Schematic of embossing manufacturing to create the EM scaffolds. The multichannel structure is fabricated as described and then assembled into the outer sheath that is sutured to the nerve. **B** The macroscopic cross-section of EM scaffolds demonstrates the multichannel packing and exhibits >70% open lumen volume. **C** The polycaprolactone (PCL) walls of the EM scaffold are highly uniform, with microporosity. Embossed scaffolds exhibited mechanical integrity and very low deformation after **D** bend testing and **E** compression testing: 1: before testing, 2: maximum loading, 3: post testing. Scale bar: **B** = 250 µm, **C** = 50 µm, **D **= 5 mm, **E** = 1 mm.

PCL multichannel scaffolds were manufactured via a novel embossing technique (specific details in “Methods”). The embossing method greatly simplified the fabrication of the multichannel scaffold, creating scaffolds with porous walls and reproducible features on the microscale that are highly aligned. Dip-coating techniques (the previous route of manufacture) previously yielded an open lumen volume of ~50 vol% (range of 45–52 vol%)^34^. Due to the reduction and increased uniformity in wall thickness (60–70 µm) using the embossing method, we achieved open lumen volumes of >70 vol% (Fig. 1B, C), thus providing more open space for guided regeneration. The change in manufacturing did not compromise the mechanical integrity of the embossed scaffolds (Fig. 1D, E). In fact, the embossed scaffolds remained highly flexible with compliances of 7.0 ± 1.5 mm/N and 42 ± 13 mm/N compression and bending, respectively. The embossed scaffolds were less likely to deform permanently, showing only 12.8 ± 2.8% change in diameter, compared to dip-coated scaffolds that deformed by 26.0 ± 1.5%^34^ (Fig. 1D, E and Supplemental Videos 1–2).

Next, we tested the extended efficacy of embossed scaffolds in a 1 cm-long sciatic nerve injury in rats^31^ (short gap injury model, mimicking the common digital nerve repair case in the clinic) and a 3 cm-long sciatic nerve injury in rabbits^35^ (mimicking a more proximal long-gap nerve repair case).

### In vivo study I: 1 cm gap injury in rats: axon regeneration into scaffolds and distal nerve after 4 weeks

Fischer 344 rats with 1 cm sciatic nerve transection injury were implanted with either (A) Multichannel scaffolds (*n* = 11), (B) Open tubes (*n* = 11), (C) Sural nerve autografts (*n* = 11).

We used an open tube device as a control because our multichannel scaffold is also considered a device by the FDA and not a biologic (Avance allograft is a biologic). We did not include an allograft group because its performance is equivalent to open tubes in gap lengths under 15 mm (RECON clinical trial^28^). We used the sural nerve autograft as a gold-standard of care for nerve gaps exceeding 1 mm.

**Fig. 2:**
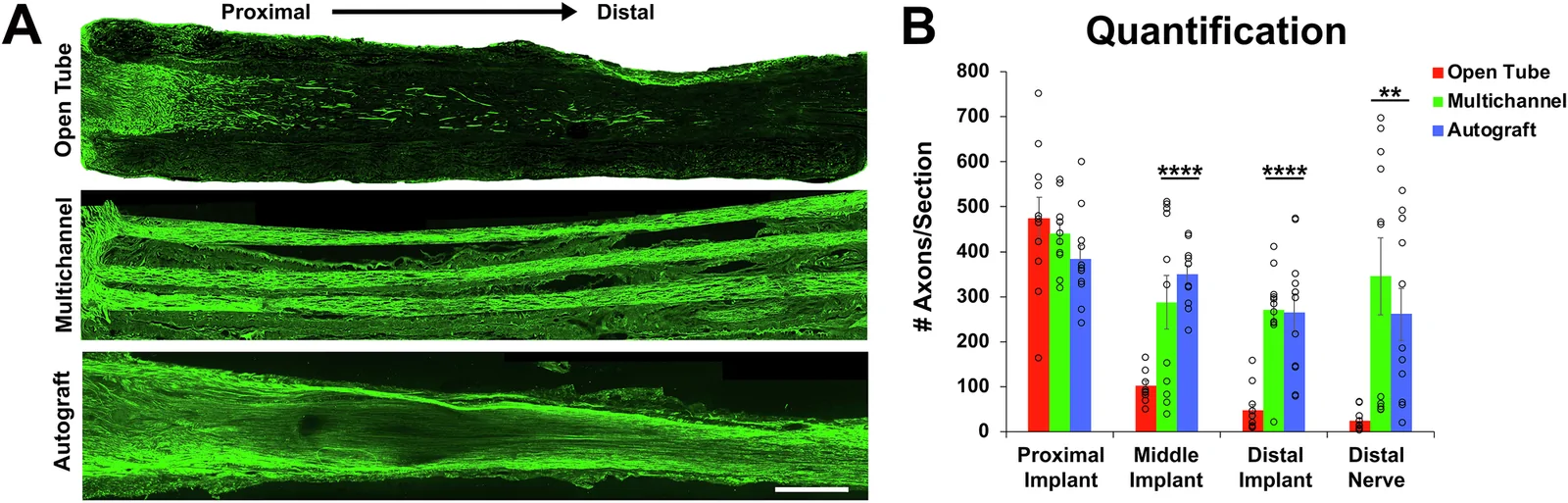
Axon regeneration across 1 cm rat sciatic gap after 4 weeks. **A** 1-cm-long injury site labeled for neurofilament 200 (NF-200, green), indicating that axons fail to bridge the injury 4 weeks after implantation of an open tube. In contrast, both the multichannel scaffold and the autograft supported complete axonal bridging across the injury. Scale bar: 1 mm. **B** Quantification of axon regeneration across 1 cm rat gap after 4 weeks. Mean ± SEM. One-way ANOVA, ****P = 0.0001, **P = 0.0018, post hoc Tukey’s, *n* = 11 animals/group.

**Fig. 3:**
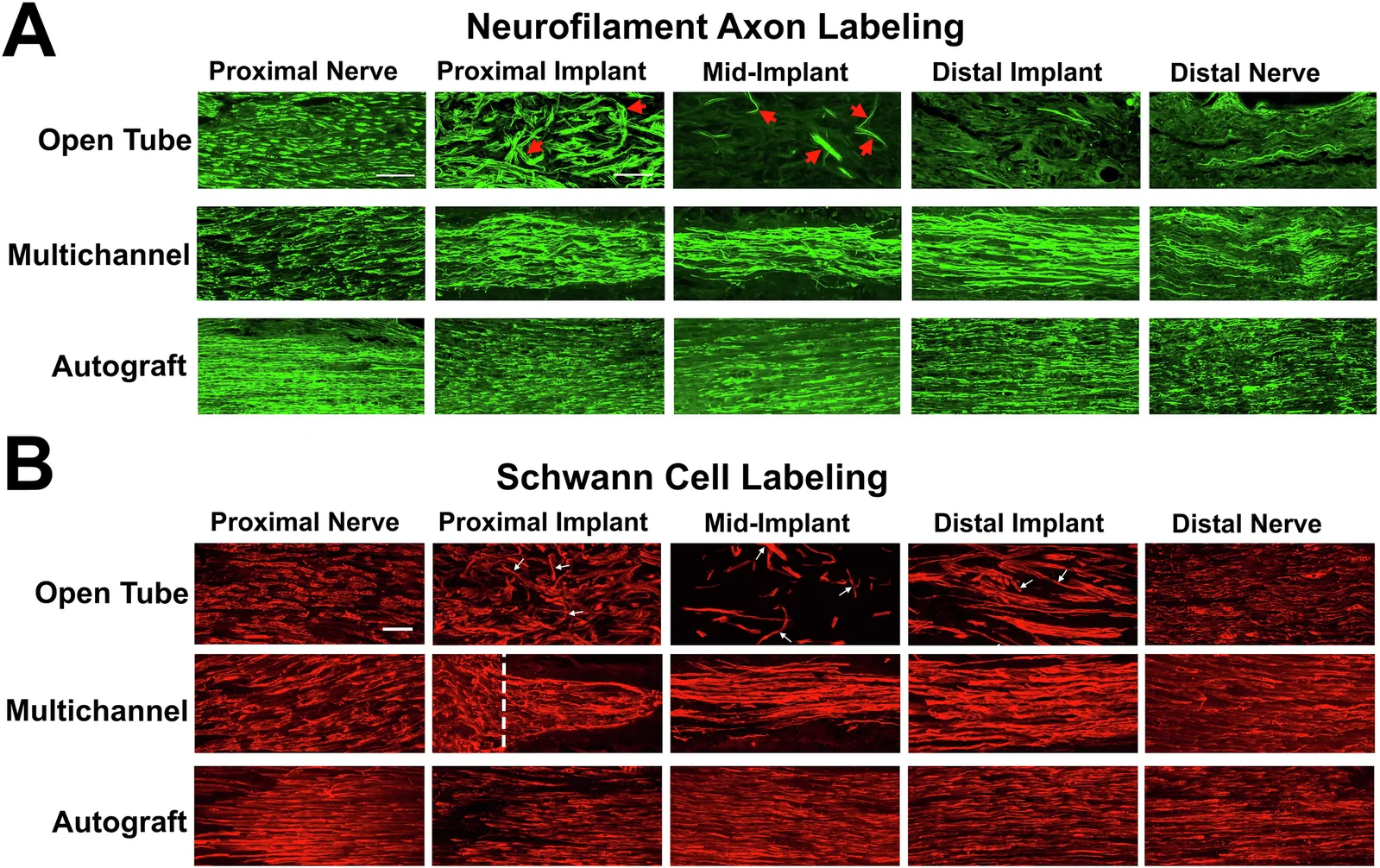
Axon regeneration across 1 cm rat sciatic gap after 4 weeks: neurofilament and Schwann cell labeling. **A** High-magnification images from the proximal nerve, through the implant, and into the distal nerve. Green: NF200 labeling for axons. Axons within open tube implants demonstrate random orientations, some turning back towards the proximal side (red arrows). Very few axons reached the distal nerve. Axons in the multichannel scaffold and autograft implant are linear and aligned in the proximal–distal axis. Many axons reached the distal nerve. Scale bar: 50 µm. **B** Red: S100 labeling for Schwann cells. Similar to axons, Schwann cells are frequently misaligned within the open tube (white arrows), unlike the linear, longitudinal orientation in the multichannel scaffold and autograft implant. Few Schwann cells are observed in the middle of the open tube, whereas many are present in the middle of the multichannel scaffold and autograft implant. The white demarcated line shows the interface between the proximal nerve and the multichannel scaffold. These high-magnification images of the multichannel scaffold show one channel out of many to show individual axon and Schwann cell alignment. Scale bar: 50 µm.

Four weeks after implantation, most axons in the open-tube group remained confined to the proximal side of the implant, whereas the majority of axons in the multichannel scaffold and autograft groups had progressed to the distal side and exited into the distal nerve (Figs. 2 and 3). Axons within the open tubes lacked linear organization and were oriented in multiple directions, with some even turning backward toward the proximal stump. In contrast, axons within multichannel scaffolds and autografts were uniformly aligned along the proximal–distal axis of regeneration (Fig. 3). These findings indicate that guiding and organizing regenerating axons accelerates axonal bridging and helps restore native nerve architecture.

Schwann cells populated all implants. In multichannel scaffolds and autografts Schwann cells were present throughout the length of scaffolds including their innermost midsections, whereas substantially fewer Schwann cells were present in the mid-region of open-tube implants (Fig. 3B). Similar to axonal regeneration patterns, Schwann cells in the open tubes were misaligned, whereas Schwann cells within the multichannel scaffolds and autografts were longitudinally oriented along the proximal–distal axis of regeneration (Fig. 3B and Supplementary Fig. 1). Quantification of Schwann cell alignment revealed that 98.0% of Schwann cells were aligned within 20° of the regeneration axis in the multichannel group (394 of 402 cells), compared to 98.3% in the autograft group (395 of 402 cells) but only 60.2% in the open tube group (242 of 402 cells). Multichannel and autograft groups differed significantly from the open tube group (*N* = 6/group, ANOVA, *P* < 0.0001; *P* < 0.0001 post hoc comparison of multichannel or autograft group to open tube group) (Supplementary Fig. 1). The multichannel group was statistically equivalent to the autograft group (post hoc *P* = 1.0). These findings indicate that both autograft and the multichannel scaffold maintain a highly organized Schwann-cell architecture along the direction of regeneration, while the absence of stricter internal guidance cues in open tube repairs results in a more random Schwann-cell orientation pattern.

Quantification of axon counts across the proximal, middle, and distal regions of the implants, extending into the distal nerve, revealed significantly greater numbers of axons in animals with multichannel scaffolds and autografts compared to open tubes (Fig. 2B). Multichannel implants contained statistically equivalent numbers of axons compared to autografts. These results demonstrate that structural guidance is essential for sustaining axonal extension across the injury: without this organization, axons and Schwann cells fail to maintain directional growth, leading to incomplete bridging. Thus, implants that recapitulate native nerve architecture markedly improve regenerative progression.

Host immune response in the nerve proximal and distal to implants was evaluated using CD45 immunolabeling (detects leukocyte subclasses including T and B cells, and granulocytes) 4 weeks post sciatic nerve injury in rats (Supplementary Fig. 2). CD45-labeled cells were present in both proximal and distal nerve segments adjacent to the implants, and all repair strategies exhibited comparable densities of CD45 immunoreactivity, indicating a similar host immune response across treatments. Previous studies have demonstrated that peripheral nerve injury initiates a coordinated immune response during which infiltrating immune system cells contribute to axonal and myelin debris clearance, Schwann cell activation, extracellular matrix remodeling, and support of axonal regeneration^36–38^. This immune response is essential to clear the nerve and injury site of debris so that successful regeneration can occur^36,37,39^. Accordingly, the comparability among groups is expected.

### In vivo study II: 1 cm gap injury in rats: axon regeneration into scaffolds and distal nerve after 6 months

Fischer 344 rats with 1 cm sciatic nerve transection injury were implanted with either (A) Multichannel scaffolds (*n* = 11), (B) Open tubes (*n* = 11), (C) Sural nerve autografts (*n* = 11), or (D) Lesion-only (*n* = 11).

**Fig. 4:**
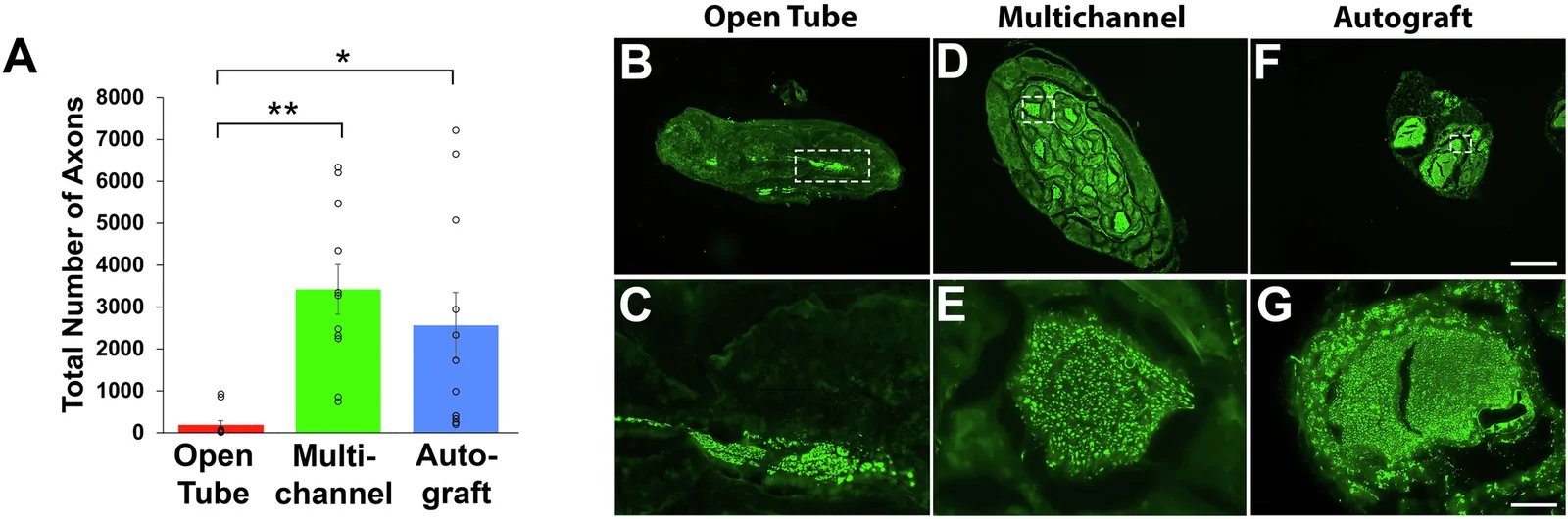
Axon regeneration into scaffolds after 6 months. **A** Significantly more axons regenerated into multichannel scaffolds compared to open tube scaffolds, while no differences in axon counts were observed when compared to the sural nerve autograft. Mean ± SEM, one-way ANOVA followed by Tukey’s post hoc test, *N* = 11 animals/group. *P = 0.017, **P = 0.0011. **B** Low-magnification image showing a representative transverse section from the center of open tube scaffolds. **C** Representative high-magnification image of axons in the open tube scaffolds. **D** Low-magnification image showing a representative transverse section from the center of the multichannel scaffolds. **E** Representative high-magnification image of axons in the multichannel scaffolds. **F** Low-magnification image showing a representative transverse section from the center of sural nerve autografts. **G** Representative high-magnification image of axons in the sural nerve autografts. Green = NF200. Scale bar: **C**, **E**, **G** = 250 µm. Scale bar: **D**, **F**, **H** = 50 µm.

We first characterized the extent of axonal regeneration within the scaffolds (Fig. 4A). Embossed multichannel scaffolds contained 3420 ± 592 axons compared to 2560 ± 791 axons in sural nerve autografts and 190 ± 104 axons in animals implanted with open tubes (Fig. 4B, D–I). The number of axons penetrating multichannel scaffolds and sural nerve autografts was significantly greater than the number of axons penetrating open tubes (Mean ± SEM. One-way ANOVA *P *< 0.0012; post hoc Tukey’s comparing multichannel scaffolds and sural nerve autografts to open tubes). The number of axons regenerating into the middle of multichannel scaffolds was 30% greater than the number of axons regenerating into sural nerve autografts, but this difference was not statistically significant (*P* = 0.5). Thus, multichannel scaffolds and sural nerve autografts support equal axonal regeneration into the mid-portion of a gap injury.

Numerous Schwann cells myelinated axons in the center (0.5 cm distance) of the multichannel scaffold (Supplementary Fig. 3). In addition, multichannel scaffolds were well vascularized (Supplementary Fig. 3).

**Fig. 5:**
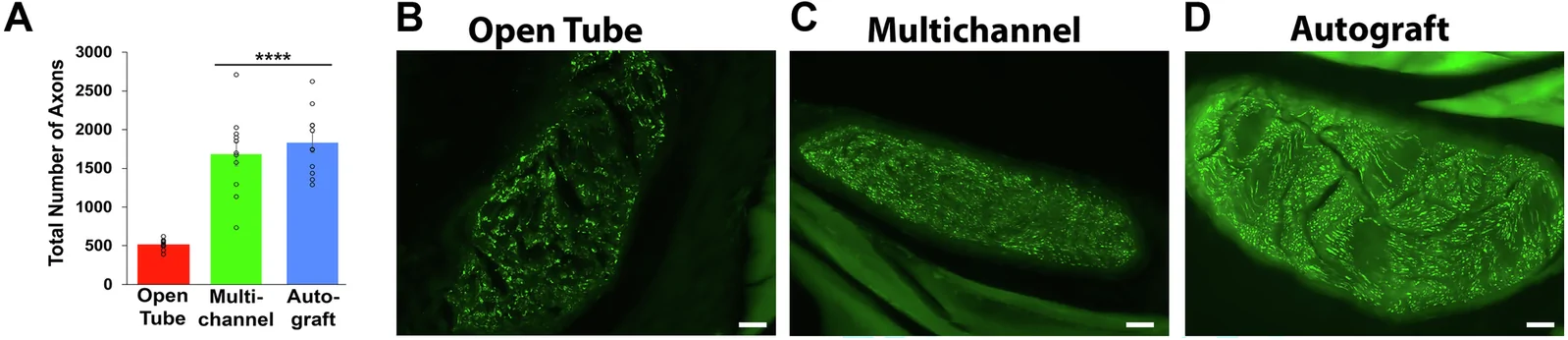
Axon regeneration at the end of the distal nerve after 6 months. **A** Significantly more axons regenerated into the gastrocnemius muscle in animals treated with multichannel scaffolds compared to open tube scaffolds, while no differences in axon counts were observed when compared to the sural nerve autograft (mean ± SEM, one-way ANOVA followed by Tukey’s post hoc test, *n* = 11 animals/group). **B** A representative transverse section showing axons at the end of the nerve at the gastrocnemius muscle from an animal treated with an open tube scaffold. **C** A representative transverse section showing axons at the end of the nerve at the gastrocnemius muscle from an animal treated with a multichannel scaffold. **D** A representative transverse section showing axons at the end of the nerve at the gastrocnemius muscle from an animal treated with sural nerve autograft. Green = NF200. Scale bar: **B**–**D** = 200 µm.

We also quantified the number of axons that bridged entirely across the lesion to reach the gastrocnemius muscle (Fig. 5). A mean of 1684 ± 156 axons were present at the end of the tibial nerve among recipients of multichannel scaffolds, 1830 ± 127 axons in recipients of sural nerve autografts, and 513 ± 18 axons in the open tube group (Fig. 5). Both the scaffold and autograft group differed significantly from the open tube group (mean ± SEM, one-way ANOVA *P* < 0.0001; post hoc Tukey’s comparing multichannel scaffolds or sural nerve autografts to open tubes). The scaffold and autograft groups did not significantly differ from each other (*P* = 0.6). In lesion-only control rats, the two transected nerve stumps were still disconnected at the end of the study; we did not quantify the number of axons in the distal nerve. The results of this experiment suggest that multichannel scaffolds and sural nerve autografts support axonal regeneration to the muscle and significantly exceed the effect of open tubes.

### Connectivity between spinal cord motor neurons and the gastrocnemius muscle

**Fig. 6:**
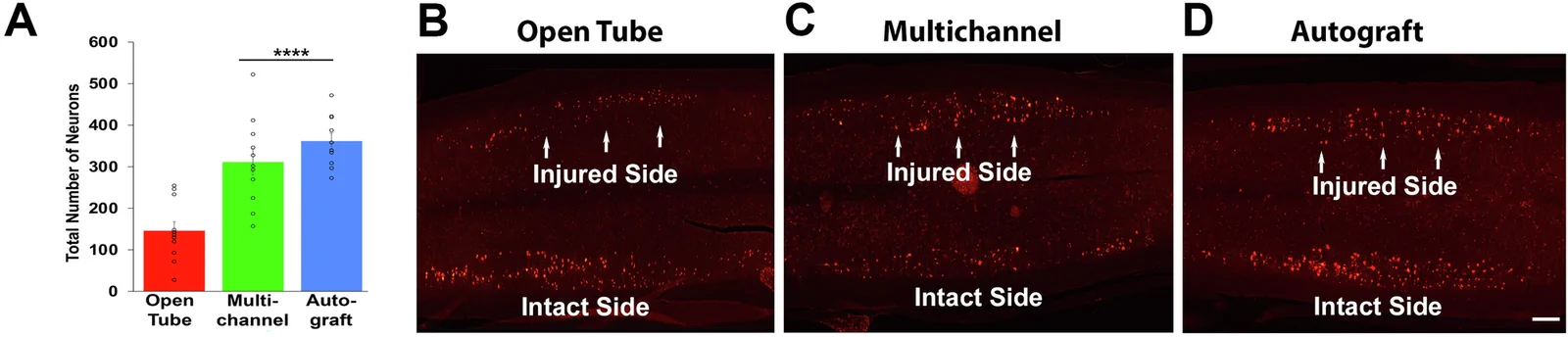
CTB-labeled motoneurons in the lumbar spinal cord. **A** Significantly more motoneurons were visible in the area representing the injured leg in the lumbar spinal cord, in both the multichannel and sural nerve autograft groups, compared with open tube scaffolds (mean ± SEM, one-way ANOVA followed by Tukey’s post hoc test, *n* = 11 animals/group. ****P = 0.0001). **B**–**D** CTB-labeled motoneurons in the ventral spinal cord of animals implanted with **B** open tube, **C** multichannel scaffold, and **D** sural nerve autograft groups. Red = CTB. Scale bar = 500 µm.

Next, we examined the extent to which connectivity was re-established between spinal cord alpha motor neurons and affected peripheral muscles among animals of the three treatment groups. Cholera toxin B (CTB) injections were placed into the gastrocnemius muscle, and the number of alpha motor neurons retrogradely labeled in the spinal cord was quantified^40^. In all, 311 ± 31 spinal motor neurons were retrogradely labeled among animals that received multichannel scaffolds compared to 361 ± 20 neurons among animals that received sural nerve autografts and 146 ± 22 neurons in animals with open tube scaffolds (Fig. 6B–E). Once again, multichannel scaffold and autograft groups exhibited significantly greater spinal cord-to-muscle connectivity compared to animals with open tubes (one-way ANOVA, *P* = 0.0001, post hoc Tukey’s), and the multichannel and autograft groups did not differ from one another (one-way ANOVA, *P* = 0.35).

### Recovery of gastrocnemius muscle mass

**Fig. 7:**
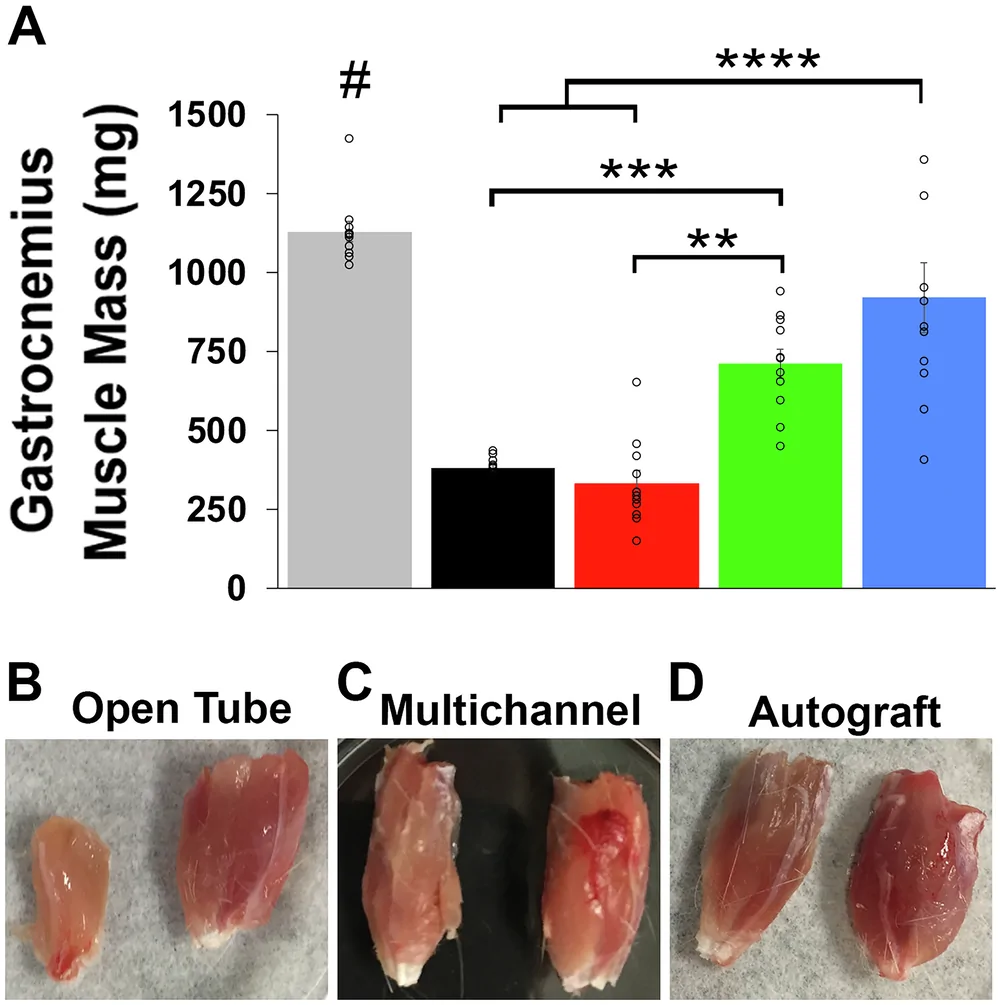
Gastrocnemius muscle mass. **A** The mass of the gastrocnemius muscles in both the multichannel and sural nerve autograft groups was significantly greater than lesion-only controls and open tube scaffolds following 6 months of regeneration. None of the groups fully regained the mass of the intact muscles at this timepoint (mean ± SEM, one-way ANOVA post hoc Tukey’s. **P = 0.0038, ***P = 0.0007, ****P = 0.00001. *n* = 11 animals/group). **B**–**D** Injured (left) and intact (right) gastrocnemius muscles from **B** open tube, **C** multichannel, and **D** *s*ural nerve autograft groups.

The mass of the gastrocnemius muscle is a functional measure of muscle reinnervation because denervation leads to muscle atrophy and loss of mass. Recipients of multichannel scaffolds and sural nerve autografts exhibited significantly heavier gastrocnemius muscles compared to open tube and lesion-only groups (Fig. 7A–D): multichannel scaffolds, 710 ± 46 mg; sural nerve autografts, 920 ± 110 mg; open tube, 330 ± 41 mg; lesion-only 380 ± 10 mg (mean ± SEM, one-way ANOVA *P* < 0.0001; post hoc Tukey’s comparing multichannel scaffolds or sural nerve autografts to open tube and lesion-only. *n* = 11 animals/group). The weight of the muscle did not differ statistically when comparing the multichannel scaffold and the autograft groups (*P* = 0.13, post hoc Tukey’s).

Neuromuscular junctions were present in the gastrocnemius muscle in animals implanted with multichannel scaffolds (Supplementary Fig. 1).

Thus, our short-gap rat study indicates that the regenerative efficacy of the multichannel scaffold is equivalent to that of an autograft. To our knowledge, this is the first demonstration of a biomimetic device achieving bioequivalence to autografts.

### In vivo study III: 3 cm gap injury in rabbits: axon regeneration into scaffolds and distal nerve after 5 months

New Zealand rabbits with 3 cm sciatic nerve transection injury were implanted with (A) Multichannel scaffolds (3 mm diameter and 3cm-long) (*n* = 11). (B) Open tube (*n* = 11). (C) Reversed sciatic nerve autograft (*n* = 11). (D) Lesion-only (*n* = 11). We used the sciatic nerve in the rabbits due to anatomical and surgical challenges in harvesting a long segment of the sural nerve, which is associated with challenges to the recovery and overall health of the animals due to the long incision in the hindlimb. We did not use Avance cadaveric allografts as a control group in this experiment because the FDA determined that allograft efficacy at longer (>25 mm) gaps has not been established^41^.

**Fig. 8:**
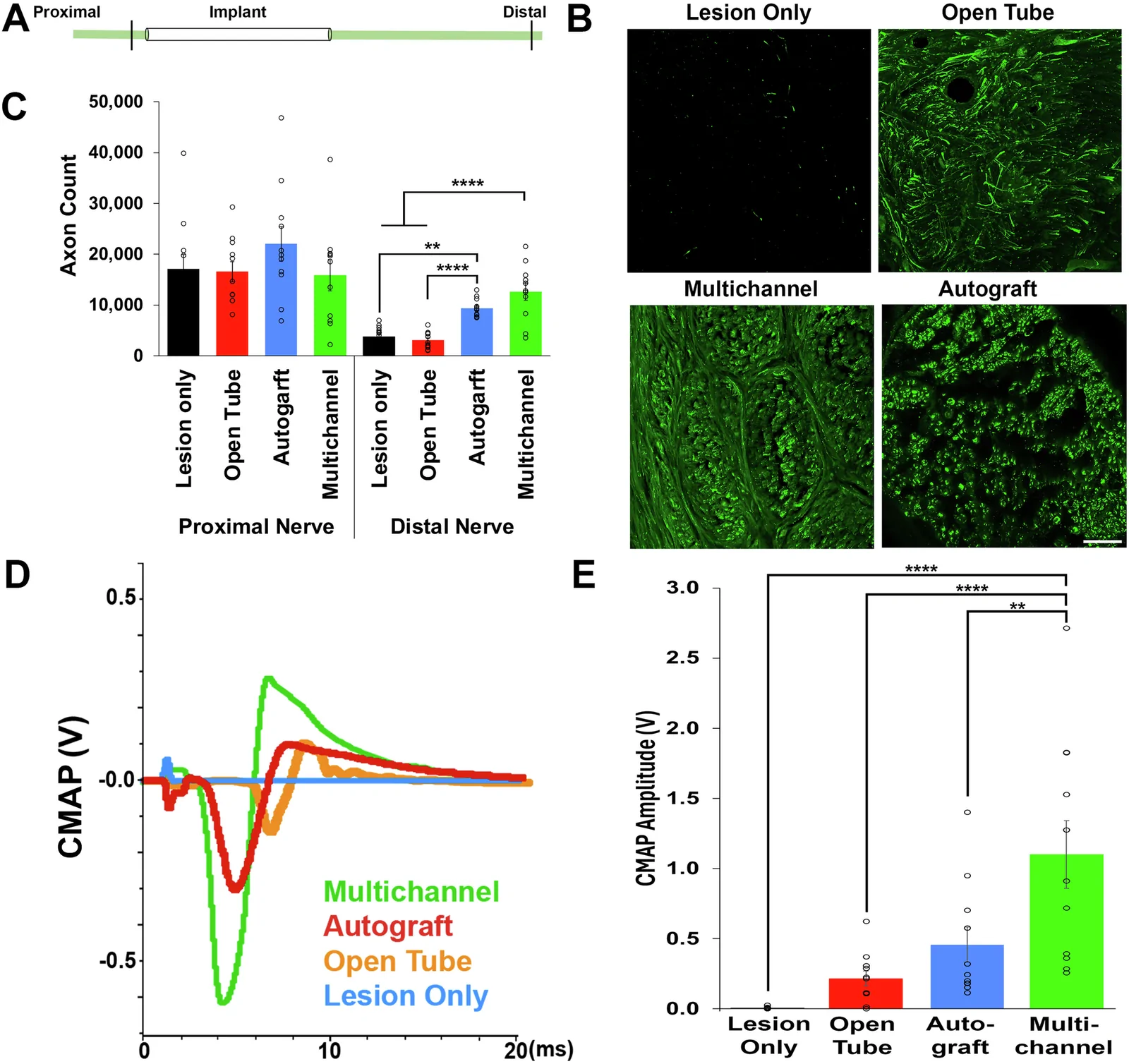
Rabbit studies. **A** We performed anatomical studies to count the number of regenerating axons through the implant site. We compared the number of axons proximal to the implant and at the distal end of the nerve as it enters the gastrocnemius muscle. **B** Transverse sections of the interface between the distal aspect of the scaffold and the distal nerve stump. Regenerating axons (green, NF200) that bridged the injury through a multichannel scaffold or an autograft were properly organized, showing the same profile. Axons regenerating through an open tube were randomly oriented in all directions. They are not positioned in the proper regeneration axis from proximal to distal. **C** We then quantified the number of regenerating axons. Animals treated with a multichannel scaffold had significantly more axons in the gastrocnemius muscle compared to injury-only and open tube treatments, but not significantly more than animals treated with an autograft (mean ± SEM, one-way ANOVA post hoc Tukey’s. ****P = 0.0001, **P = 0.0012, *n *= 11 animals/group). **D**, **E** Animals treated with multichannel scaffolds recovered higher amplitudes of CMAPs compared to all other treatments. Mean ± SEM, one-way ANOVA *P* = 0.0001; post hoc Tukey’s **P = 0.01 compared to autograft, ****P = 0.0003 compared to open tube, and ****P = 0.0001 compared to lesion-only. *n* = 11 animals/group.

We first characterized the extent of axonal regeneration across the gap and into the tibial nerve, which innervates the gastrocnemius muscle (Fig. 8A). In rabbits that were part of the injury-only group, the nerve stumps were connected with fibrous tissue, and we therefore included these subjects in the quantification studies, unlike the injury-only group in the rat studies. We examined the interface between the distal end of the repair and the distal nerve stump. Notable differences in the spatial organization of regenerating axons were observed across the treatment groups. In both the multichannel scaffold and autograft groups, regenerating axons that bridged the injury site maintained a consistent orientation along the proximal–distal axis. In contrast, axons that regenerated through an open tube exhibited more heterogeneous spatial orientation and were randomly distributed throughout the tissue, indicative of misalignment (Fig. 8B).

We then quantified the number of regenerating axons. As expected, axon numbers were similar across all groups in the proximal nerve (one-way ANOVA, *P* = 0.4; Fig. 8C). Notably, in the tibial nerve segment distal to the repair we found the highest numbers of axons in animals treated with multichannel scaffolds (12,590 ± 1669) compared to 9390 ± 563 axons in animals treated with autografts, 3070 ± 498 axons in animals that received open tubes, and 3810 ± 658 axons in injury-only subjects. Overall differences between groups were significant (Fig. 8C, mean ± SEM, one-way ANOVA post hoc Tukey’s. *P* = 0.0001, *n* = 11 animals/group), with multichannel and autograft groups differing significantly from lesion-only and open tube groups. The higher number of axons in the multichannel scaffold trended toward a difference from the autograft group but did not reach statistical significance (*P *= 0.15, post hoc Tukey’s). Thus, multichannel scaffolds and autografts exhibit superiority to open tubes and lesion-only groups.

### Recovery of compound muscle action potentials

We performed a terminal electrophysiological study to characterize electrical conductivity across the 3 cm nerve gap. The sciatic nerve was stimulated proximal to each type of implant, and we recorded compound motor action potentials (CMAPs) using gastrocnemius muscle EMG implants (Fig. 8D, E). Notably, the greatest degree of recovery occurred in subjects that received multichannel scaffolds, exhibiting statistical superiority to all other groups (ANOVA *P* = 0.0001; post hoc Tukey’s *P* = 0.01 compared to autograft, *P* = 0.0003 compared to open tube, and *P* = 0.0001 compared to lesion-only (Fig. 6E). The magnitude of CMAP recovery was twofold greater than in the autograft group: multichannel scaffold mean CMAP amplitude was 1.1 ± 0.24 V; autograft, 0.45 ± 0.12 V; open tube, 0.21 ± 0.05 V; and injury-only, 0.0076 ± 0.0022 V (mean ± SEM). Thus, in longer gap injuries, multichannel scaffolds outperformed autografts in electrophysiological recovery measures.

Thus, our long-gap rabbit study indicates that the regenerative efficacy of the multichannel scaffold is at least equivalent to that of an autograft, and on some measures is superior. To our knowledge, this is the first demonstration that a biomimetic device achieves bioequivalence or superiority to autografts at long gap lengths.

#### Gross histology

There were no gross inflammatory reactions to any of the implant types (Supplementary Fig. 4A). H&E stains of multichannel scaffolds demonstrated robust vascularization and absence of major inflammatory responses (Supplementary Fig. 4B).

## Discussion

FDA-approved devices for peripheral nerve repair have several limitations compared to autografts, including donor size mismatch and, most importantly, inferior efficacy of repair^23,42–46^. Autografts also have limitations, including added surgical time for donor harvesting, numbness, and sometimes, dysaesthesia in the donor nerve distribution, and an approximate 20% incidence of chronic pain in the donor site^9^. The goal of our scaffold design was to overcome these limitations and create a synthetic scaffold that spans nerve gaps and facilitates nerve regeneration to an extent that equals the efficacy of autografts, allowing use of the device for nerve repair instead of autografts. Our approach focused on creating a scaffold that mimics the fascicular architecture of the intact nerve, thereby organizing regenerating axons and improving their spatial organization. Through a novel manufacturing process that efficiently and reproducibly fabricates microchannels out of PCL sheets, we created a device with microtubules of consistent dimensions that achieves a cross-sectional area available for axonal regeneration of 70% and has mechanical integrity and is biocompatible. The embossed scaffold microchannels provide highly linear guidance to injured axons, demonstrating the ability to direct axons through the injury gap to the distal nerve in aligned linear arrays similar to their pre-injury orientation. Modifications to the scaffold diameter and length can be readily controlled, allowing the manufacture of larger and smaller diameter scaffolds of various lengths to accommodate virtually any nerve diameter and gap length. The versatility, ease of fabrication, and multichannel structure of these scaffolds overcome many of the key limitations of current FDA-approved devices for peripheral nerve repair.

While the beneficial mechanical and structural properties of these porous PCL scaffolds are advantageous over current scaffold designs, the main goal is to promote regeneration that compares to nerve autografts used clinically. Our short-term 4-week study and long-term six-month rat studies demonstrated that embossed multichannel scaffolds organized regenerating axons in a manner that mimics the normal fascicular architecture of the intact nerve. These features resulted in statistically equivalent outcomes to autografts anatomically and functionally, as reflected in the quantification of regenerating axon numbers, motor neuron-to-muscle connectivity, and muscle weight. Remarkably, at long gap distances of 3 cm in a rabbit model, multichannel scaffolds exhibited statistically equivalent support of axonal regeneration compared to autografts and significantly *exceeded* autograft efficacy, resulting in more than a twofold improvement in electrophysiological muscle response recovery.

Schwann cell markers demonstrated extensive Schwann cell fill of multichannel scaffolds and autografts that aligned along the long axis of the nerve, thereby supporting and guiding axonal regeneration. Immune or inflammatory responses to multichannel scaffolds were not observed, perhaps unsurprisingly, since the primary material composition of the implants is poly-caprolactone (PCL), the material from which many types of sutures are manufactured.

In our rabbit study, our primary functional outcome measure assessed EMG function in the muscle rather than a behavioral test such as the sciatic functional index or toe-spread. Both the sciatic functional index and toe spread are less reliable in rabbits because they frequently develop foot complications that can mechanically restrict leg and digit extension regardless of the actual degree of functional regeneration^47–49^. Moreover, rabbits are highly sensitive and reactive to handling in the experimental setting, resulting in substantial variability in behavioral outcome measures and poor interobserver reliability. Consequently, *objective* measurements such as electrophysiological recordings to quantify motor reinnervation / functional recovery are considered more reliable and are widely used functional endpoints^47–50^. Compound muscle action potential (CMAP) amplitude reflects the extent of motor axon reinnervation of the target muscle and correlates with the number of regenerated axons, making it an objective indicator of functional recovery that is also used clinically to evaluate peripheral nerve recovery^50–52^.

When an autograft is introduced into a nerve gap injury, the resident cells and axonal debris must first die off and subsequently become phagocytosed, a process that requires several weeks. In contrast, multichannel scaffolds can support immediate host Schwann cell and vascular migration into the gap, followed rapidly by host axonal penetration. Particularly over longer gap injuries, prolonged times for initiation of regeneration can result in distal Schwann cell atrophy and reduced capacity to secrete neurotrophic factors^53–55^. In addition, the multichannel design provides 70% open space for axonal regeneration, whereas in human donor nerves (autografts or allografts) the percent open area is only 33.1% in the sural nerve^7^ and 21% in the terminal branch of the posterior interosseous nerve^56^; these are the most common nerves used for autografts or allografts. The remaining proportion of nerve is connective tissue and fat. The presence of an organized and strictly linear growth substrate for host cell migration and axonal regeneration, with a high percentage of area that is open for regeneration, may account for the superior performance of multichannel scaffolds compared to autografts at longer gap lengths in our study.

Why did we not compare our multichannel scaffold to decellularized cadaveric allografts such as AxoGen’s Avance? In preparing for an IDE clinical trial application to the FDA, we were required to compare our scaffold to a similarly manufactured device: a cadaveric allograft is considered a biological product rather than a device and is therefore not suitable for an FDA comparative submission. However, we can compare the performance of our multichannel scaffold in this preclinical study to previously published preclinical studies of cadaveric allografts^23,24,57–61^. (A) A recent meta-analysis of 50 animal studies predominantly employing rat models found that processed allografts were significantly inferior to autografts in seven of eight histological and functional outcomes, including muscle weight, Sciatic Function Index, ankle angle, nerve conduction velocity, axon count, axon diameter, and tetanic contraction force^57^. (B) Whitelock et al demonstrated that a rat nerve treated with the AxoGen decellularization process, in order to generate an allograft for implant, exhibited distorted fascicular architecture that differs significantly from the natural architecture of the intact nerve^23^; this structural degradation may be one factor contributing to the inferior performance of allografts to autografts in preclinical models. (C) Across most studies, cadaveric allografts performed inferiorly to autografts^23,24,57–61^. These findings are consistent with results of the cadaveric allograft RECON clinical trial in which allografts performed no better than the Neuragen tube, which contains only a single open channel^28^. In contrast, our preclinical study demonstrates statistical equivalence or superiority of our multichannel scaffolds to autografts on all preclinical outcome measures.

Many different designs for peripheral nerve repair devices and allografts have been studied in nerve injury models^21,23,24,62–65^. However, to the best of our knowledge, there are no devices that: (1) perform as well as or better (based on the injury size) than nerve autografts, and (2) can be readily modified to meet nerve diameter and injury length constraints. We demonstrated that embossed multichannel scaffolds promote axon regeneration at levels similar to or superior to nerve autografts, providing an off-the-shelf alternative for efficient and safe nerve repair. These devices are now undergoing testing in a prospective, randomized, and controlled multicenter clinical trial in peripheral nerve gap injury (NCT06529835) compared to open tube conduits and autografts.

## Methods

### Implant preparation

#### Polymer films

Films were cast by pouring the PCL/salt slurry onto copper foil (McMaster) and spreading using an automated tape casting coater (MTI Corporation), with the adjustable blade set to 500 μm clearance. Films were air-dried and detached from the copper by wetting in ethanol and stored at room temperature until use. Prior to embossing, the film (PCL + salt) is pressed using a calendar (Hohsen Corp. heated desktop calendar roll press), in sequential stages, to a final thickness of 80–90 µm.

#### Open tubes

Open tubes are made by dipping a stainless-steel rod having a 1.6-mm outer diameter into the polymer + salt slurry. Once dry, the coated rod is immersed in ethanol, and the tube is formed by sliding the PCL + salt tube off the rod.

#### Embossing materials preparation

Stainless-steel wires (280 µm outer diameter, California Fine Wires) were prepared, as described before^34^. Wires, cleaned in ethanol, are dipped into a solution of 12 wt% poly(vinyl alcohol) (PVA) in water, pre-heated to 60 °C. Coated wires were dried and then inspected by eye for drips or inhomogeneities in the coating. If drips or inhomogeneities in the coating are found, the wires are excluded from manufacturing. An acetal embossing mold (DELRIN) is machined to have linear grooves separated by 135 µm. Each groove is made with a 330 µm endmill, resulting in features that are ~300 µm wide and ~150 µm deep. A mold for pressing the final multichannel roll together is constructed from aluminum. The mold comprised two pieces registered to produce a single channel of 1.6-mm diameter when fit together.

A schematic of the embossing process is presented in Fig.1A. To prepare for embossing, two aluminum blocks and a pressing mold were heated to 40–50 °C. The embossing mold was used at temperatures between 21 °C and 60 °C. Sheets of 2-mm foam (Darice) are cut to the size of the embossing mold. Two squares of pressed film (e.g., produced as described above) were cut to dimensions that result in a scaffold of the desired lateral length and number of channels. An initial film was laid on the embossing mold and covered by a piece of foam, placed between the pre-heated aluminum blocks, and compressed. Stainless-steel wires, pre-coated with a 12 wt% poly(vinyl alcohol) (PVA) solution^34^, were placed in the grooves on the embossing mold and secured in place with adhesive. Sixteen wires produced a 1.6-mm diameter device. The second film was placed over the top of the wire array, aligned to match the area of the first embossed film, and covered by a second piece of foam. The sandwich was placed between the aluminum blocks and pressed with at least 1 metric ton of force for 20 s. To form a scaffold, the embossed film with wire spacers is removed from the embossing block. The embossed film was rolled around an axis parallel to the axis of the spacers. The roll was placed into a pressing mold, pre-heated to 40–50 °C. This mold was placed between the two aluminum blocks and pressed to 1.5 metric tons of pressure. Once removed from the press, the mold was opened, and the roll was removed as a finished bundle of microchannels. The roll was then inserted into an open tube, formed via dip-coating, and the assembly was immersed in deionized water to remove the salt and PVA coating. The stainless-steel spacers were removed, and the EM scaffold was air-dried.

### Mechanical testing

All mechanical testing was performed as described previously^34^. Prior to mechanical testing, all scaffolds were incubated in PBS at 37 °C overnight. Scaffolds were removed from the liquid just prior to testing at room temperature. All mechanical tests were run with 4–5 samples.

### Compression

Scaffolds (10 mm length, 1.5 mm diameter) were tested in transverse compression (perpendicular to the long axis). A TA XT plus texture analyzer was used to compress the scaffolds at a rate of 1 mm/min to 60% strain, then the scaffolds were unloaded at 1 mm/min. The entire cycle was repeated to assess deformation. Compliance, a measure of how easily the material could be compressed, was computed from the inverse slope of the linear region of a Force-displacement curve, constructed from the first loading cycle. The percentage of deformation was calculated as the percent change in diameter of the scaffold after the initial load cycle. This was measured as the distance the tester had to travel to achieve 0.02 N load on the sample during the second cycle.

### Imaging

#### Scanning electron microscopy (SEM)

Pre-hydrated scaffolds were frozen in liquid nitrogen and cross-sectioned with a razor blade. Slices were placed on an aluminum stub for microscopy, and sputter-coated with gold for 4 min at 40 mA. Imaging was performed on a Hitachi S-3500N, operating at 20 keV and a working distance of 10 mm.

### Surgical techniques

#### Rat studies

Adult female F344, 200 g, 3-month-old rats were used in this study. All animal studies were carried out according to NIH guidelines for laboratory animal care and safety, adhering to protocols approved by the Institutional Animal Care and Use Committee of the Veterans Administration Health System, San Diego. Rats were allowed unrestricted movement after recovery and were housed with access to food and water ad libitum in VMU rooms maintained at constant temperature (19–22 °C) and humidity (40–50%) on a 12:12 h light/dark cycle. Animals were deeply anesthetized (ketamine (25 mg ml^−1^), xylazine (1300 mg ml^−1^), and acepromazine (0.25 mg ml^−1^), then a 20 mm long skin incision was made on the left lateral thigh, and the left sciatic nerve trunk was exposed via a lateral gluteal muscle dissection. Epineural connective tissue surrounding the nerve trunk was separated with microscissors, and the nerve trunk was exposed approximately 20 mm from the level of the obturator externus tendon. A 10.0 mm long nerve segment was excised with sharp microscissors and replaced with implants attached at either end using 9-0 Ethicon sutures. Care was taken to avoid tension at the interfaces of the scaffold and nerve site. Groups: PCL open tube (*n* = 11), PCL embossed multichannel scaffold (*n* = 11). For the sural nerve autograft group (*n* = 11), the sural nerve was exposed from the branching point of the sciatic nerve to the most distal part near the tendon. It was excised and cut into two 1 cm-long cables. Both cables were implanted into the injured sciatic nerve using 9-0 Ethicon sutures. For the lesion-only group (*n* = 11), no implant was used to bridge the lesion site. Following implantation, muscles were sutured using a 5-0 suture, and the skin was closed using wound clips. Antibiotics and analgesics (banamine (1 mg kg^−1^) and ampicillin (0.2 mg kg^−1^) in Ringer’s lactate) were administered for the first 3 days to facilitate recovery from surgery.

#### CTB injections

Three days prior to sacrifice animals were anesthetized using isoflurane, a 3 mm medial incision was made in the posterior leg, exposing the gastrocnemius muscle. A 2.5 µL injection of 1% Alexa Fluor 594 conjugated, cholera toxin subunit B (CTB – ThermoFisher) was made into the left gastrocnemius muscle (injured side), to label reconnected motor neurons in the lumbar spinal cord.

#### Determination of muscle mass

Following perfusion, both gastrocnemius muscles were removed and weighed using a microbalance. Intact muscles were averaged across all animals.

#### Rabbit studies

Adult New Zealand White rabbits (Western Oregon Rabbitry, Philomath, OR), 3.5 kg each, were used in this study. All animal studies were carried out according to NIH guidelines for laboratory animal care and safety, adhering to protocols approved by the Institutional Animal Care and Use Committee of the Veterans Administration Health System, San Diego. Rabbits were allowed unrestricted movement after recovery and were housed with access to food and water ad libitum in VMU rooms maintained at constant temperature (19–22 °C) and humidity (40–50%) on a 12:12 h light/dark cycle. Animals were given a subcutaneous injection of ketamine (30 mg/kg) in the subscapular region for anesthetic induction. Once induced, animals were masked on isoflurane under a slow induction up to 2.5% isoflurane before being intubated with Rusch® uncuffed Murphy Endotracheal tubes (Teleflex®, Morrisville, NC). Animal vitals were monitored using a Surgivet® Advisor® Vital Signs Monitor (Model V9203, Smiths Medical, Dublin, OH) throughout the procedure. Animals were positioned in left lateral recumbency, and an approximately 8 cm long incision was performed over the lateral surface of the right thigh. Blunt dissection was used to expose the right sciatic nerve from proximal to distal bifurcations. The nerve was cut distally before the bifurcation with sharp scissors and allowed to retract. After this, a ruler was used to measure 3 cm, and the proximal end was cut to create a 3 cm gap. At this point, the injury site was either closed (*n* = 11), or was microsurgically repaired with a multichannel scaffold (*n* = 11), an open tube scaffold (*n* = 11), or a reversed sciatic nerve autograft (*n* = 11), using 8-0 Ethilon suture (Ethicon, Somerville, NJ). The muscles and skin were closed using 3-0 Vicryl (Ethicon).

#### Electrophysiological studies

We used our electrophysiology setup as was reported before^66^ with the following modifications. We used a hook electrode to stimulate the sciatic nerve proximal to the implant and a needle electrode to record from the gastrocnemius muscle. Recordings were performed as a terminal study on the day of perfusion. Repeatable recorded potentials were obtained and averaged.

#### Tissue processing and immunohistochemistry

Animals were perfused with 4% cold paraformaldehyde (PFA), tissue (implant, distal sciatic nerve, gastrocnemius muscle, and spinal cord) was removed and post-fixed in 4% PFA for additional 24 h followed by 48 h in 30% sucrose. Implants and the distal sciatic nerve were sectioned transversely; the spinal cord and the gastrocnemius muscle were sectioned horizontally using a cryostat. All sections were 35 µm thick.

Spinal cord sections were coverslipped without any further processing. Implant, distal sciatic nerve, and gastrocnemius muscle sections were incubated overnight at 4 °C with the primary antibody mouse monoclonal NF200 (1:500, Millipore MAB5262), or rabbit myelin basic protein (MBP; 1:500, Dako A0623), or mouse monoclonal RECA-1 (1:500, ABCAM ab9774), or CD45 Biotin (1:500, Miltenyi 130-117-199), or Alexa Fluor 555 Alpha-Bungarotoxin (1:500, ThrmoFisher Scientific B35451) followed by incubation with Alexa 488 conjugated donkey anti mouse (1:250, Jackson Laboratories 714454150), or Alexa 568 Donkey anti rabbit (1:250, Jackson Laboratories 711575152), or Streptavidin Alexa 647 (1:250, Invitrogen 521374), or Alexa 568 Donkey anti mouse (1:250, Invitrogen A10037) secondary antibodies overnight at 4 °C, and then coverslipped. Eight sections per animal were quantified. Quantification was performed using ImageJ. CD45 immunofluorescence intensity was quantified in ImageJ using ×40 magnification images acquired on an Olympus fluorescence microscope under identical exposure and gain settings across all samples.

Schwann-cell alignment was quantified from fluorescence micrographs using automated image analysis. Schwann cells ≥25 pixels in length were identified, and their orientation was measured relative to the longitudinal regeneration axis. To ensure representative sampling across each image, equal numbers of cells were selected from the top, middle, and bottom thirds of each image. Total cell counts were subsequently equalized across treatment groups prior to analysis. Cellular orientation was analyzed as axial data and visualized using circular histograms with 20° bins. Cells were classified as aligned when their orientation was within 20° of the regeneration axis. The proportion of aligned cells was compared among groups using a chi-square test followed by pairwise Fisher exact tests with Holm correction for multiple comparisons.

#### Statistics and reproducibility

Multiple group comparisons were tested using one-tailed ANOVA (JMP Pro 16) at a designated significance level of *P* < 0.05, followed by post hoc analysis using Tukey’s test. The scaffold compression studies used four scaffolds, each as a separate replicate. The scaffold bending studies used five scaffolds, each as a separate replicate. In vivo data were collected from one rat study with *n* = 11 animals per group and one rabbit study with *n* = 11 animals per group.

### Ethics

Every experiment involving animals has been carried out following a protocol approved by VA San Diego Institution Animal Care and Use Committee.

### Reporting summary

Further information on research design is available in the Nature Portfolio Reporting Summary linked to this article.

## Supplementary information

Supplementary Information

Description of Additional Supplementary Files

Supplemental Video 1

Supplemental Video 2

Reporting Summary

Transparent Peer Review file

## Source data

Source data

## Data Availability

All data supporting the findings of this study are available within the article and its supplementary files. Data supporting the findings of this study are available from ykoffler@health.ucsd.edu. All requests for materials and data are promptly reviewed by the Office of Innovation and Commercialization at the University of California, San Diego, to verify whether the request is subject to intellectual property or confidentiality obligations. Any materials and data that can be shared will be released via a Material Transfer Agreement. Source data are provided with this paper.
