# Supplementary material for: Multichannel, biomimetic scaffolds improve functional connectivity following traumatic long-gap peripheral nerve injury
Source: Nat Commun. 2026 Sep 21;17:11015. doi: 10.1038/s41467-026-77537-6 (PMC13687026; doi:10.1038/s41467-026-77537-6)
Supplement: Supplementary file 1 [file 41467_2026_77537_MOESM1_ESM.pdf]

## Supplementary Information

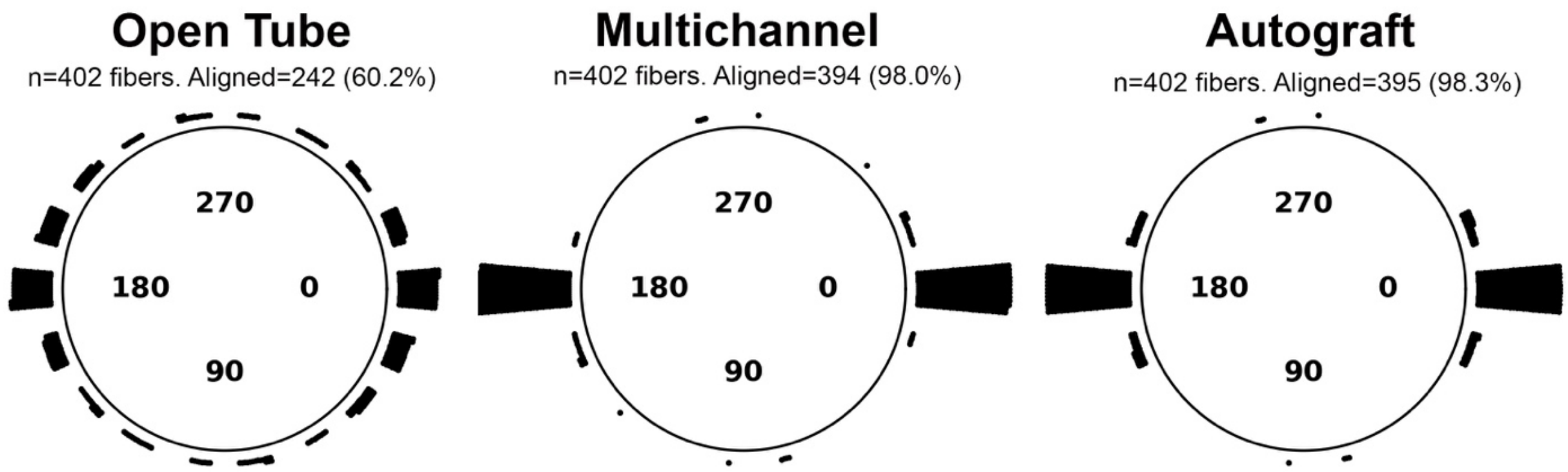


**Supplemental Figure 1: Schwann cell organization following peripheral nerve repair.** Circular histograms showing Schwann-cell orientation in the open tube, multichannel and autograft groups. Cellular orientation was measured relative to the longitudinal regeneration axis (proximal to distal; left to right in S100 labeled images). Dots represent individual cells grouped into 20° bins. The inner circle indicates the reference orientation axis (0°, 90°, 180°, and 270°). A total of 402 cells were analyzed per group. The proportion of aligned fibers (<20° from the regeneration axis) differed significantly among groups ($p < 0.0001$). Pairwise comparisons demonstrated significantly greater alignment in the autograft and multichannel groups compared with open tube repair (adjusted $p < 0.0001$ for both comparisons), whereas no significant difference was detected between autograft and multichannel repair (adjusted $p = 1.0$) n=6 animals/group.

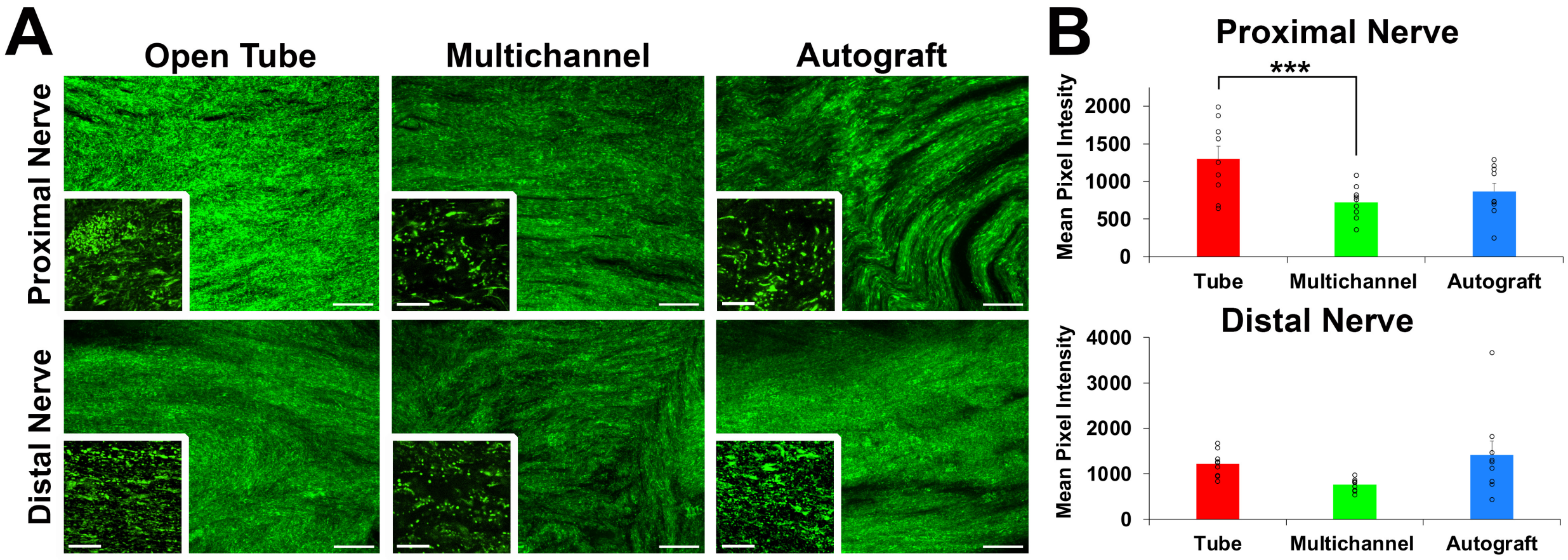


**Supplemental Figure 2: Immune response in nerve tissue surrounding the implants, 4 weeks post sciatic nerve injury in rats. A.** Representative longitudinal sections immunolabeled for CD45 (green) in proximal and distal nerve segments following repair with an open tube, multichannel device or autograft. Insets show higher magnification. Scalebar: 100µm and 10 µm (insets). **B.** Quantification of CD45 mean fluorescence intensity. Similar levels of CD45 immunoreactivity were observed among groups overall, in both distal and proximal nerve tissue, although a modest increase in proximal CD45 fluorescence intensity was detected in the Open Tube group compared to the Multichannel group (Mean ±SEM one-way ANOVA followed by Tukey's post hoc test, $p=0.008$, $n=9$ animals/group). Thus, the multichannel scaffold does not activate an immune response differing from the other treatment conditions.

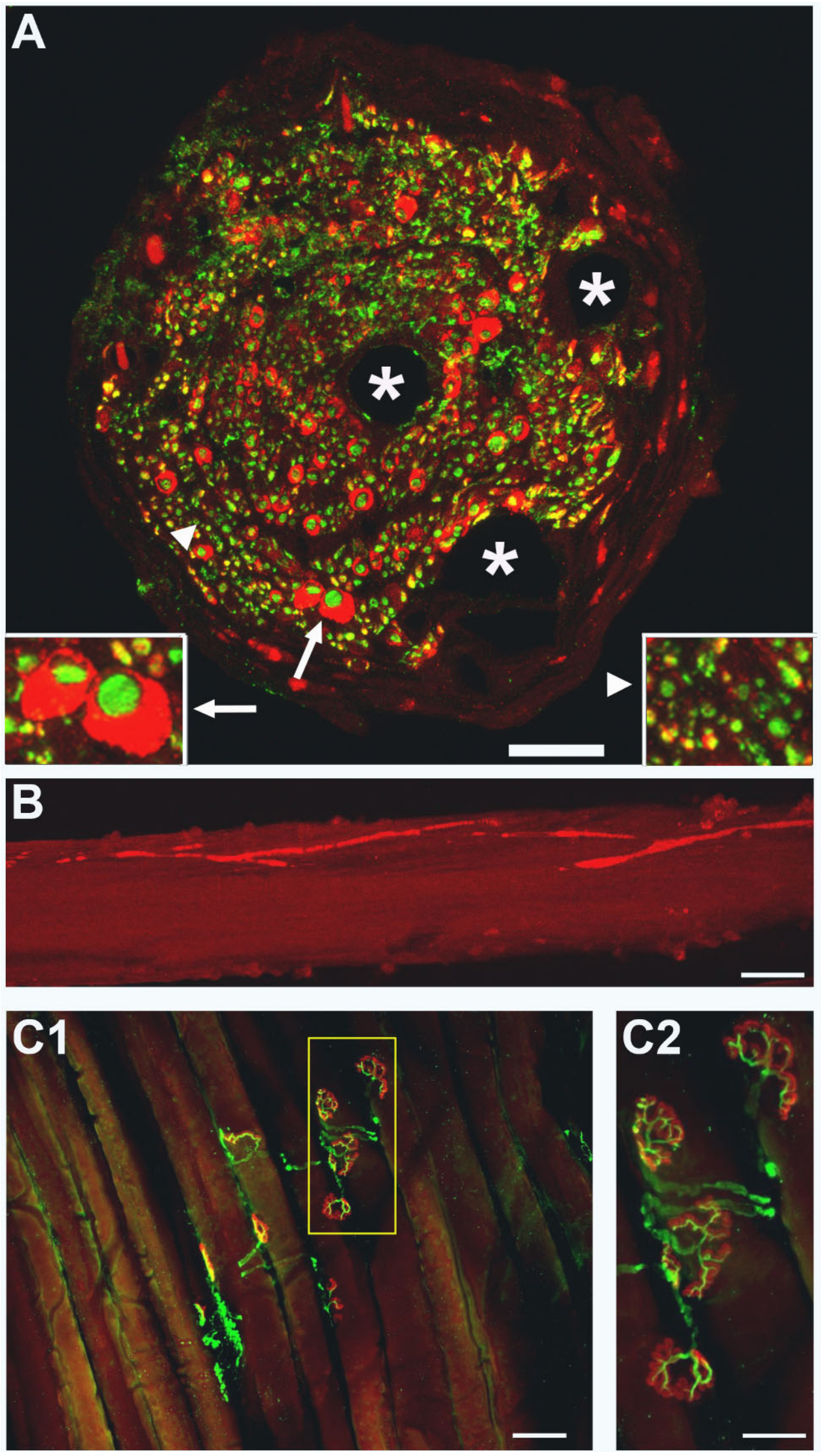


**Supplemental Figure 3: A.** High magnification cross sectional image of one channel in multi-channel scaffold. Many myelinated (**MBP-red**) axons (**NF200-green**) are present in the middle of a 1cm-long multichannel scaffold. White arrow points to larger axons with thicker myelin sheaths, and arrowhead points to region of smaller unmyelinated axons. Asterisks indicate blood vessels. Longitudinal section. **B.** RECA-1 labeled blood vessels (red) in the center of a 1cm-long multichannel scaffold. **C1.** 6 months post implantation, neuromuscular junction (NMJ) innervation was observed in the gastrocnemius muscle among animals that received scaffolds. **NF200** labeled axons in **green** and α bungarotoxin labeled **NMJ**s in **red**. **C2.** High magnification image from yellow box in C1 showing axons innervating NMJs. Scale bar: A-20μm. B-C1: 50μm. C2. 20μm.

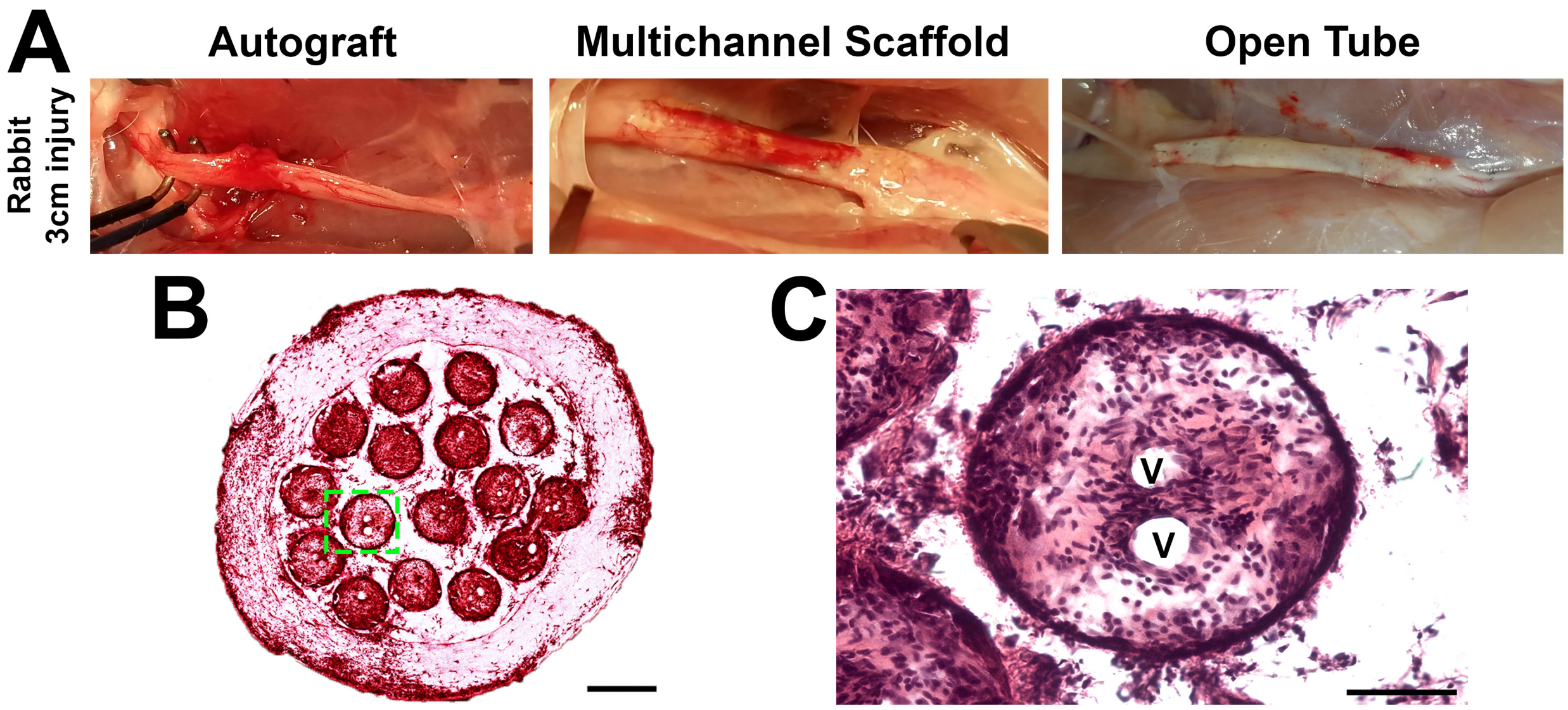


**Supplemental Figure 4**: **A.** Macroscopic view of implantation sites 5 months post-transection in rabbit 3cm gap injury. No gross scarring is observed at the implantation sites, and surrounding muscle tissue exhibits no evidence of toxicity. **B.** Low magnification view of H&E stain of multichannel scaffold in rat, showing excellent multichannel architecture over 6 months. 35μm section. **C.** Higher magnification view of single channel from panel B demonstrates blood vessels in scaffold center and absence of major inflammatory response. Scale Bar B 500 μm; C 100 μm.
