# Supplementary material for: Multichannel, biomimetic scaffolds improve functional connectivity following traumatic long-gap peripheral nerve injury
Source: Nat Commun. 2026 Sep 21;17:11015. doi: 10.1038/s41467-026-77537-6 (PMC13687026; doi:10.1038/s41467-026-77537-6)
Supplement: Supplementary file 2 [file 41467_2026_77537_MOESM2_ESM.pdf]

<u>Description of Additional Supplementary Files</u>

Supplementary Video 1: Compression test showing device performance before testing, during maximum load and post testing.

Supplementary Video 2: Bending test showing device performance before testing, during maximum load and post testing.
