# Supplementary material for: Multichannel, biomimetic scaffolds improve functional connectivity following traumatic long-gap peripheral nerve injury
Source: Nat Commun. 2026 Sep 21;17:11015. doi: 10.1038/s41467-026-77537-6 (PMC13687026; doi:10.1038/s41467-026-77537-6)
Supplement: Supplementary file 3 [file 41467_2026_77537_MOESM5_ESM.pdf]

nature portfolio

Corresponding author(s): Jacob Koffler, PhD, MBA, Department of Neuroscience, University of California San Diego, La Jolla, CA, USA 92093 (ykoffler@ucsd.edu)

Last updated by author(s): Aug 24, 2026

Reporting Summary

Nature Portfolio wishes to improve the reproducibility of the work that we publish. This form provides structure for consistency and transparency in reporting. For further information on Nature Portfolio policies, see our Editorial Policies and the Editorial Policy Checklist.

Statistics

For all statistical analyses, confirm that the following items are present in the figure legend, table legend, main text, or Methods section.

n/a	Confirmed	
☐	☒	The exact sample size (*n*) for each experimental group/condition, given as a discrete number and unit of measurement
☐	☒	A statement on whether measurements were taken from distinct samples or whether the same sample was measured repeatedly
☐	☒	The statistical test(s) used AND whether they are one- or two-sided<br>*Only common tests should be described solely by name; describe more complex techniques in the Methods section.*
☐	☒	A description of all covariates tested
☒	☐	A description of any assumptions or corrections, such as tests of normality and adjustment for multiple comparisons
☐	☒	A full description of the statistical parameters including central tendency (e.g. means) or other basic estimates (e.g. regression coefficient) AND variation (e.g. standard deviation) or associated estimates of uncertainty (e.g. confidence intervals)
☐	☒	For null hypothesis testing, the test statistic (e.g. *F*, *t*, *r*) with confidence intervals, effect sizes, degrees of freedom and *P* value noted<br>*Give P values as exact values whenever suitable.*
☒	☐	For Bayesian analysis, information on the choice of priors and Markov chain Monte Carlo settings
☒	☐	For hierarchical and complex designs, identification of the appropriate level for tests and full reporting of outcomes
☒	☐	Estimates of effect sizes (e.g. Cohen's *d*, Pearson's *r*), indicating how they were calculated

Our web collection on statistics for biologists contains articles on many of the points above.

Software and code

Policy information about availability of computer code

Data collection	N/A
Data analysis	N/A

For manuscripts utilizing custom algorithms or software that are central to the research but not yet described in published literature, software must be made available to editors and reviewers. We strongly encourage code deposition in a community repository (e.g. GitHub). See the Nature Portfolio guidelines for submitting code & software for further information.

Data

Policy information about availability of data

All manuscripts must include a data availability statement. This statement should provide the following information, where applicable:

- Accession codes, unique identifiers, or web links for publicly available datasets
- A description of any restrictions on data availability
- For clinical datasets or third party data, please ensure that the statement adheres to our policy

Data supporting the findings of this study are available from ykoffler@ucsd.edu on reasonable request. All requests for materials and data are promptly reviewed by the Office of Innovation and Commercialization—University of California San Diego to verify whether the request is subject to any intellectual property or confidentiality obligations. Any materials and data that can be shared will be released via a Material Transfer Agreement.

Research involving human participants, their data, or biological material

Policy information about studies with human participants or human data. See also policy information about sex, gender (identity/presentation), and sexual orientation and race, ethnicity and racism.

Reporting on sex and gender	N/A
Reporting on race, ethnicity, or other socially relevant groupings	N/A
Population characteristics	N/A
Recruitment	*Describe how participants were recruited. Outline any potential self-selection bias or other biases that may be present and how these are likely to impact results.*
Ethics oversight	N/A

Note that full information on the approval of the study protocol must also be provided in the manuscript.

Field-specific reporting

Please select the one below that is the best fit for your research. If you are not sure, read the appropriate sections before making your selection.

☒ Life sciences ☐ Behavioural & social sciences ☐ Ecological, evolutionary & environmental sciences

For a reference copy of the document with all sections, see nature.com/documents/nr-reporting-summary-flat.pdf

Life sciences study design

All studies must disclose on these points even when the disclosure is negative.

Sample size	A sample size of 11 subjects/group was determined by power analysis: 11 animals per group, standard deviation of 0.15, and effect size of 0.24 yields computed power of 0.85. This design is sufficient to determine significant group differences .
Data exclusions	None
Replication	Rat and Rabbit studies were performed once each.
Randomization	animals were assigned randomly into experimental groups.
Blinding	investigators were blinded during data collection and analyses.

Reporting for specific materials, systems and methods

We require information from authors about some types of materials, experimental systems and methods used in many studies. Here, indicate whether each material, system or method listed is relevant to your study. If you are not sure if a list item applies to your research, read the appropriate section before selecting a response.

Materials & experimental systems

n/a	Involved in the study	
☐	☒	Antibodies
☒	☐	Eukaryotic cell lines
☒	☐	Palaeontology and archaeology
☐	☒	Animals and other organisms
☒	☐	Clinical data
☒	☐	Dual use research of concern
☒	☐	Plants

Methods

n/a	Involved in the study	
☒	☐	ChIP-seq
☒	☐	Flow cytometry
☒	☐	MRI-based neuroimaging

Antibodies

Antibodies used	Primary antibody mouse monoclonal NF200 (1:500, Millipore MAB5262), or rabbit myelin basic protein (MBP; 1:500, Dako A0623), or mouse monoclonal RECA-1 (1:500, ABCAM ab9774), or CD45 Biotin (1:500, Miltenyi 130-117-199), or Alexa Fluor 555 Alpha-

	Bungarotoxin (1:500, ThrmoFisher Scientific B35451) followed by incubation with Alexa 488 conjugated donkey anti mouse (1:250, Jackson Laboratories 714454150), or Alexa 568 Donkey anti rabbit (1:250, Jackson Laboratories 711575152), or Streptavidin Alexa 647 (1:250, Invitrogen 521374), or Alexa 568 Donkey anti mouse (1:250, Invitrogen A10037) secondary antibodies
Validation	Antibodies are purchased from known reliable suppliers. Antibodies that have previously been validated in the literature are preferred. For immunohistochemistry, antibodies are confirmed by staining of fixed frozen tissue sections, performed by an experienced histology technician. Controls include immunolabeling of tissue from knockout animals for the target protein where available, and western blotting to confirm molecular weight and specificity of immunoreactive protein bands.

Animals and other research organisms

Policy information about studies involving animals; ARRIVE guidelines recommended for reporting animal research, and Sex and Gender in Research

Laboratory animals	Adult female F344 rats, 200gr each, 3 months old. . Adult New Zealand White rabbits , 3.5kg each
Wild animals	N/A
Reporting on sex	There is no evidence that clinically important sex/gender and race/ethnicity differences are to be expected; that is, there are no known differences in rate of axonal regeneration or scaffold tolerability.
Field-collected samples	N/A
Ethics oversight	The study protocol was approved by San Diego VA IACUC committee.

Note that full information on the approval of the study protocol must also be provided in the manuscript.

Plants

Seed stocks	N/A
Novel plant genotypes	N/A
Authentication	N/A
