# Supplementary material for: Multichannel, biomimetic scaffolds improve functional connectivity following traumatic long-gap peripheral nerve injury
Source: Nat Commun. 2026 Sep 21;17:11015. doi: 10.1038/s41467-026-77537-6 (PMC13687026; doi:10.1038/s41467-026-77537-6)
Supplement: Supplementary file 4 [file 41467_2026_77537_MOESM6_ESM.pdf]

nature portfolio

Peer Review File

Multichannel, Biomimetic Scaffolds Improve Functional Connectivity Following Traumatic Long-Gap Peripheral Nerve Injury

Corresponding Author: Dr Jacob Koffler

This manuscript has been previously reviewed at another journal. This document only contains information relating to versions considered at Nature Communications.

This file contains all reviewer reports in order by version, followed by all author rebuttals in order by version.

Version 0:

Reviewer comments:

Reviewer #1

(Remarks to the Author)
I agree that the degree of regeneration achieved comparable to the autograft is still very good and is the more important advance here.
Overall, the authors in my view could have gone a bit further here by providing behavioural data at least on motor function. The CMAP is good but does not show if there is a motor improvement.
Next, the characterisation of the cellular microenvironment around the graft is very limited. Supp Fig. 1 should be supported by quantification (a couple ion nice images are not sufficient). Quantitative assessment of the immune response would also be recommendable as it provides some further insight into the mechanisms, which remain unclear.

In summary, the major limitation I see in my view to take this to the clinic is the lack of data on functional recovery (motor function at least, ideally both motor and sensory, in rats this is easy and very standard

Reviewer #2

(Remarks to the Author)
I reviewed this manuscript previously and was enthusiastic about it. I remain enthusiastic and think the authors have addressed nearly all of the reviewers concerns. I have one comment that I think needs some clarification. The authors were asked previously to demonstrate that axons in the multichannel implant are myelinated. They provide S100 labeling and suggest it indicates myelination. This is not quite true. S100 labels all Schwann cells - whether myelinated or not. I think the authors need to show a mature myelin marker (e.g. MBP) or even better, they should show an electron micrograph cross section through the three cases (autograft, multi-channel, etc.). Even toluidine blue labeling of cross sections showing mature myelin would satisfy this reviewer.

Version 1:

Reviewer comments:

Reviewer #1

(Remarks to the Author)
Further to the lack of neurological assessment (that is standard in rat or mouse mdoels), the authors have not provided any quantitative measurement of NMJ formation/density in the their experimental groups. They have also ignored my suggestion to characterize the immune response (which can be easily performed with standard immunofluorescence) and they have not provided any quantitative assessment of SC organisation around the regenerating axons in their experimental groups.

Reviewer #2

(Remarks to the Author)
The authors have satisfied all of my concerns. This is a really great advance and will no doubt have an immediate impact in the clinic.

Version 2:

Reviewer comments:

Reviewer #1

(Remarks to the Author)
Supp Figure 1: it is unclear how many independent biological replicates (animals) were used for the analysis: the authors state “A total of 402 cells were analyzed per group”, however, statistical analysis shall be performed between experimental groups considering biological replicates (each one with an X number of cells that were counted). These details need to be provided.
I cannot see representative images reflecting the graphs.

Supp Figure 2: the authors used a general immune cell marker, CD45, which does not allow drawing biologically significant conclusions since all immune cells are CD45 positive. It is also not clear how many biological replicates were used and if the statistical analysis was based on independent biological replicates.

Supp Figure 4: as above: It is not clear how many biological replicates were used and if the statistical analysis was based on independent biological replicates.

ALL figures should clearly show individual data points as currently bar graphs remain unclear as to N of biological/technical replicates

Open Access This Peer Review File is licensed under a Creative Commons Attribution 4.0 International License, which permits use, sharing, adaptation, distribution and reproduction in any medium or format, as long as you give appropriate credit to the original author(s) and the source, provide a link to the Creative Commons license, and indicate if changes were made.
In cases where reviewers are anonymous, credit should be given to 'Anonymous Referee' and the source.
The images or other third party material in this Peer Review File are included in the article’s Creative Commons license, unless indicated otherwise in a credit line to the material. If material is not included in the article’s Creative Commons license and your intended use is not permitted by statutory regulation or exceeds the permitted use, you will need to obtain permission directly from the copyright holder.
To view a copy of this license, visit https://creativecommons.org/licenses/by/4.0/

We thank the reviewers for their comments and provide the following responses:

Reviewer #1

1. **Overall, the authors in my view could have gone a bit further here by providing behavioural data at least on motor function. The CMAP is good but does not show if there is a motor improvement.**

Response: Following sciatic nerve injury, rabbits frequently develop complications including pressure sores, foot trauma, and autotomy, which can mechanically restrict leg and digit extension regardless of the actual degree of functional regeneration. In addition, rabbits are highly sensitive animals, and behavioral outcomes are strongly influenced by the animal's motivation, handling, and stress levels during testing, resulting in substantial variability and poor inter-observer reliability. Consequently, subjective functional scoring systems in rabbits often fail to accurately reflect the true extent of functional nerve regeneration, highlighting the need for objective measurements such as electrophysiological recordings to quantify motor reinnervation and functional recovery.

Accordingly, in rabbit peripheral nerve injury models, electrophysiological assessment is the most widely used functional endpoint and is generally regarded as the most reliable objective measure of nerve regeneration. Compound muscle action potential (CMAP) provides an objective, quantifiable, and physiologically direct measure of motor pathway restoration, as it depends on successful conduction through regenerated motor axons to the target muscle and the resulting muscle activation. The amplitude reflects the extent of motor axon reinnervation of the target muscle and correlates with the number of regenerated axons, making it a robust indicator of functional recovery.

2. **Next, the characterisation of the cellular microenvironment around the implant is very limited. Supp Fig. 1 should be supported by quantification (a couple of nice images are not sufficient). Quantitative assessment of the immune response would also be recommendable as it provides some further insight into the mechanisms, which remain unclear.**

Response:
A. Mechanism – We agree that quantification of relevant outcomes is important. The likely mechanism of recovery after peripheral nerve transection is reconnection of the proximal nerve segment to the distal nerve segment, or more precisely, reconnection of the spinal alpha motor neuron to the neuromuscular junction. We provide quantification of number of alpha motor neurons in the spinal cord that are connected to the neuromuscular junction after injection of retrograde tracer into the muscle (Figure 6), and demonstrate the statistical and numerical superiority of this precise outcome measure in our scaffold group and the autograft group. We also quantify axon numbers regenerating across the gap and into the distal nerve (Figures 3, 4 and 5). These measures of reconnection are the mechanism most likely to account for recovery.

It is indeed important to understand the cellular microenvironment in the scaffold. We now provide images demonstrating Schwann cells entirely occupying the gap in all 3 groups (open tube, multichannel scaffold and autograft), and note the precisely linear orientation of Schwann cells only in multichannel and autograft groups (Figure 3). Moreover, the open tube group shows gaps in Schwann cell fill that are not present in the multichannel and autograft groups, particularly at the mid-portion of the gap (Figure 3). We have modified the manuscript to highlight these observations more clearly.

The purpose of Supplemental Figures 1A–B was to provide additional examples of the microenvironment within the implant. Along these lines, we have added labeling for blood vessels in new Supplementary Figure 2, together with myelin and formation of motor end plates (alpha bungarotoxin labeling).

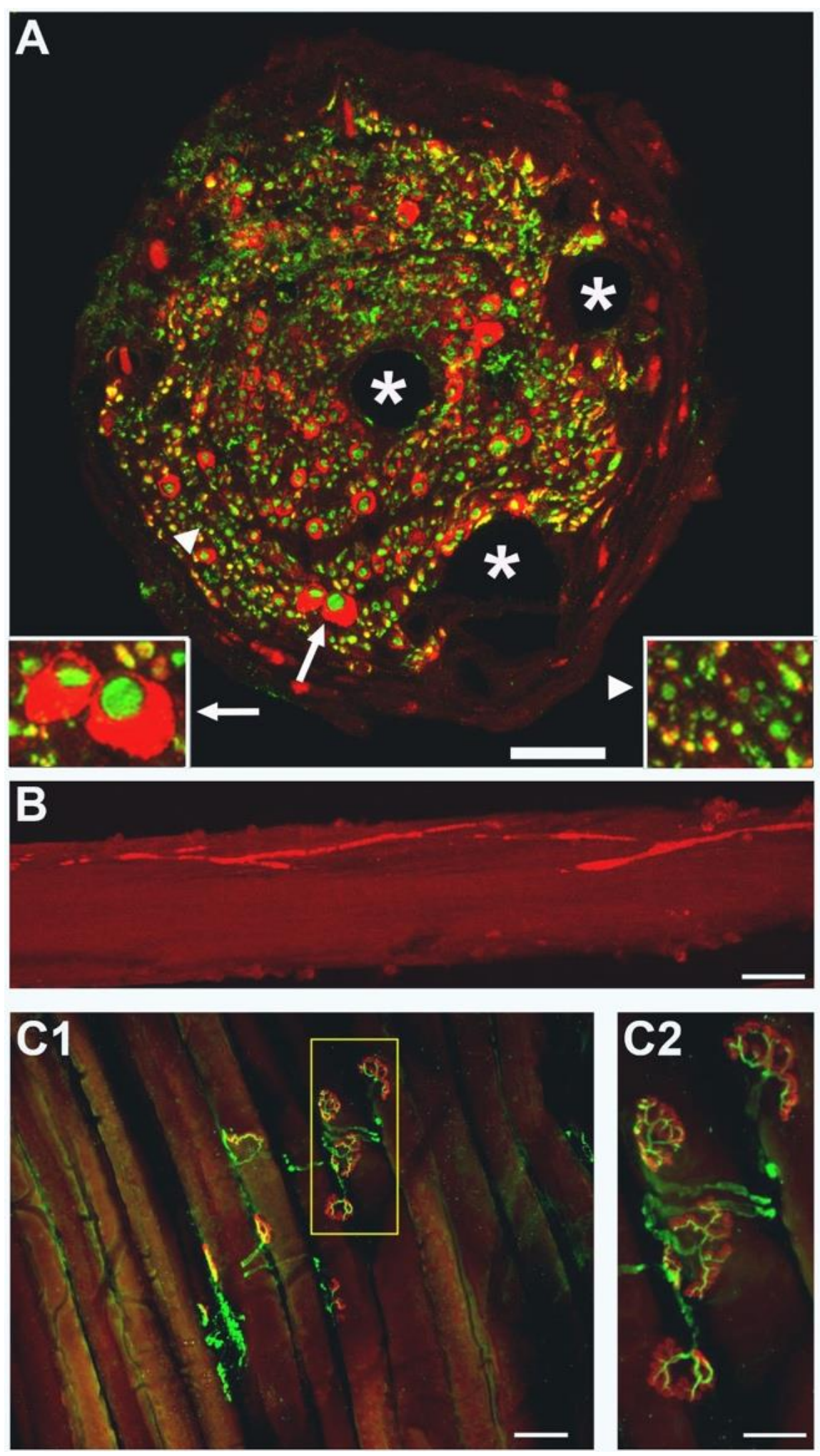

Supplemental Figure 1: A. Myelin basic protein (**MBP**) immunolabeling of a single channel in the multichannel scaffold; neurofilament is **green**. Numerous myelinated axons are visible in the channel, with the largest axons exhibiting the thickest myelin sheaths (arrow and left inset). Smaller axons are unmyelinated (arrowhead and right inset). Image from middle of a 1cm-long multichannel scaffold. **B.** RECA-1 labeled blood vessels (red) in the middle of a 1cm-long multichannel scaffold. **C1.** 6 months post

implantation, neuromuscular junction (NMJ) innervation was observed in the gastrocnemius muscle among animals that received scaffolds. NF200-labeled axons in **green** and α bungarotoxin labeled NMJs in **red**. **C2.** Higher magnification image from yellow box in C1 showing axons forming NMJs. Scale bar: A-20μm. B-C1: 50μm. C2. 20μm.

B. Immune response – Gross views of the scaffolds together with H&E stains of the scaffold have been added as new Supplementary Fig 2. Low magnification gross images demonstrate no scarring around implants (open tube, multichannel scaffold and autograft). H&E of the multichannel scaffold highlights robust vascularization and absence of major inflammatory response.

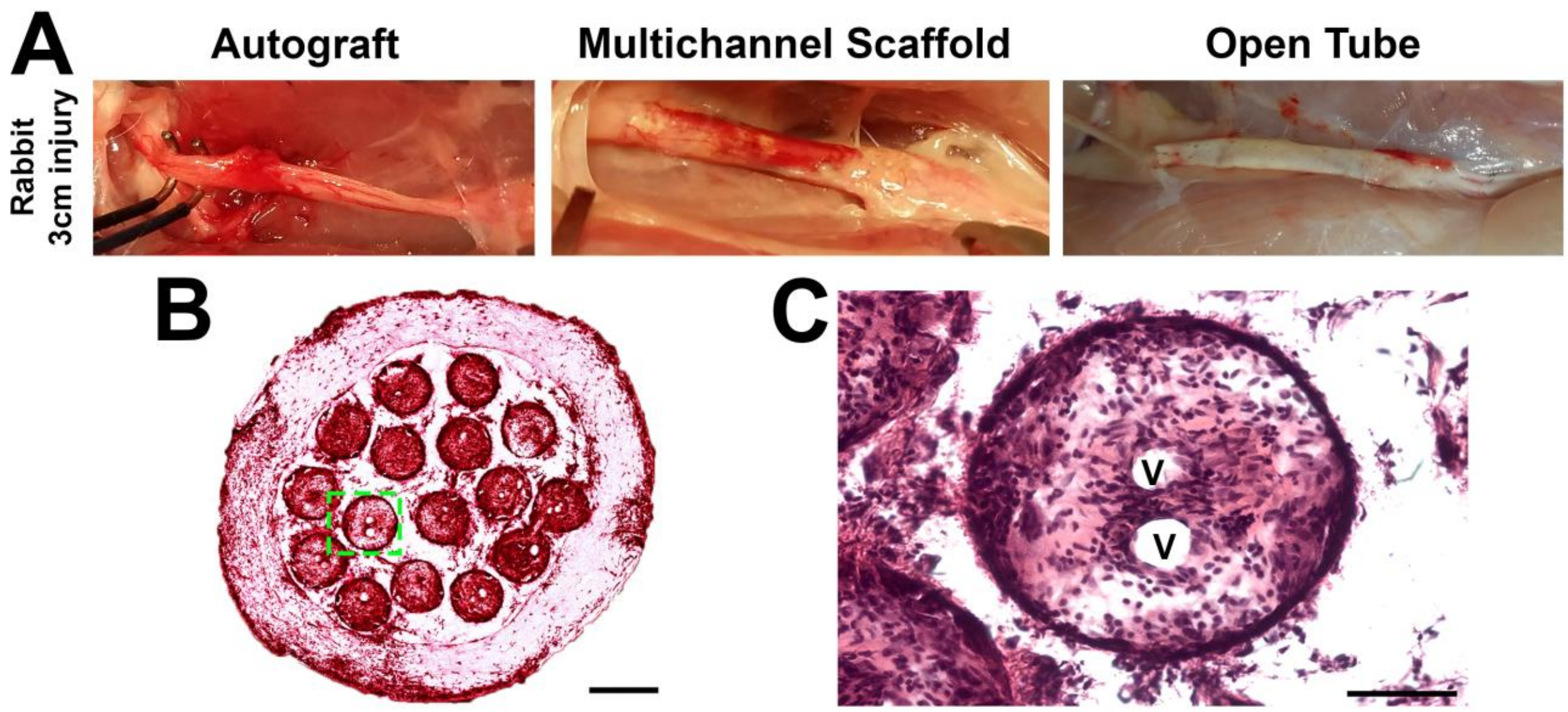

Supplemental Figure 2: **A.** Macroscopic view of implantation sites 5 months post-transection in rabbit 3cm gap injury. No gross scarring is observed at the implantation sites, and surrounding muscle tissue exhibits no evidence of toxicity. **B.** Low magnification view of H&E stain of multichannel scaffold in rat, shows retention of gross structure over 6 months. 35μm section. **C.** Higher magnification view of single channel from panel B demonstrates blood vessels in scaffold center and absence of major inflammatory response. Scale Bar B 500 μm; C 100 μm.

<u>Reviewer #2</u>

I think the authors need to show a mature myelin marker (e.g. MBP) or even better, they should show an electron micrograph cross section through the three cases (autograft, multi-channel, etc.). Even toluidine blue labeling of cross sections showing mature myelin would satisfy this reviewer.

Response: We performed labeling for myelin basic protein (MBP) and have added this to new Suppl Fig 1A. This demonstrates myelination of regenerating axons in the center of the scaffold, with thickest myelin sheaths around the largest axons and no myelin

around small diameter axons.

We are grateful for the reviewers' comments and hope that these changes are satisfactorily.

Sincerely,

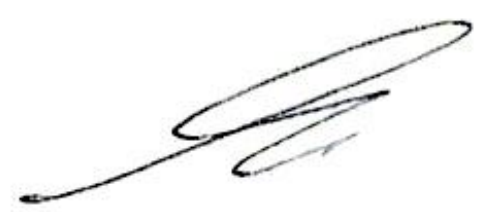

Jacob Koffler PhD MBA
Assistant professor of Neurosciences
School of Medicine, UC San Diego

We thank the reviewers for their comments and provide the following responses, which we believe represent complete responses to remaining issues identified by both reviewers.

Reviewer #1

1. Quantitative assessment of Schwann cell alignment

Response: We assessed Schwann cell alignment, characterizing their spatial organization relative to the regeneration axis from proximal to distal direction in the nerve gap. Schwann-cell alignment differed substantially among treatment groups. Both the multichannel scaffold and autograft groups demonstrated highly organized Schwann-cell alignment along the regeneration axis, whereas Schwann cells in the open tube group were more randomly oriented (Figure below and new **Supp. Fig. 1)**. In the multichannel group, 394 of 402 fibers (98.0%) were aligned within 20° of the regeneration axis, compared with 395 of 402 fibers (98.3%) in the autograft group but only 242 of 402 fibers (60.2%) in the open tube group. The proportion of aligned fibers differed significantly among groups ($p<0.0001$) (**Supp. Fig. 1)**. Pairwise analyses demonstrated significantly greater alignment in both the multichannel and autograft groups compared with the open tube ($p<0.0001$ for both comparisons), whereas no significant difference was detected between the multichannel and autograft groups ($p=1.0$). These findings indicate that both autograft and the multichannel scaffold maintain a highly organized Schwann-cell architecture along the direction of regeneration, while the absence of stricter internal guidance cues in tube repairs results in a more random Schwann-cell orientation pattern.

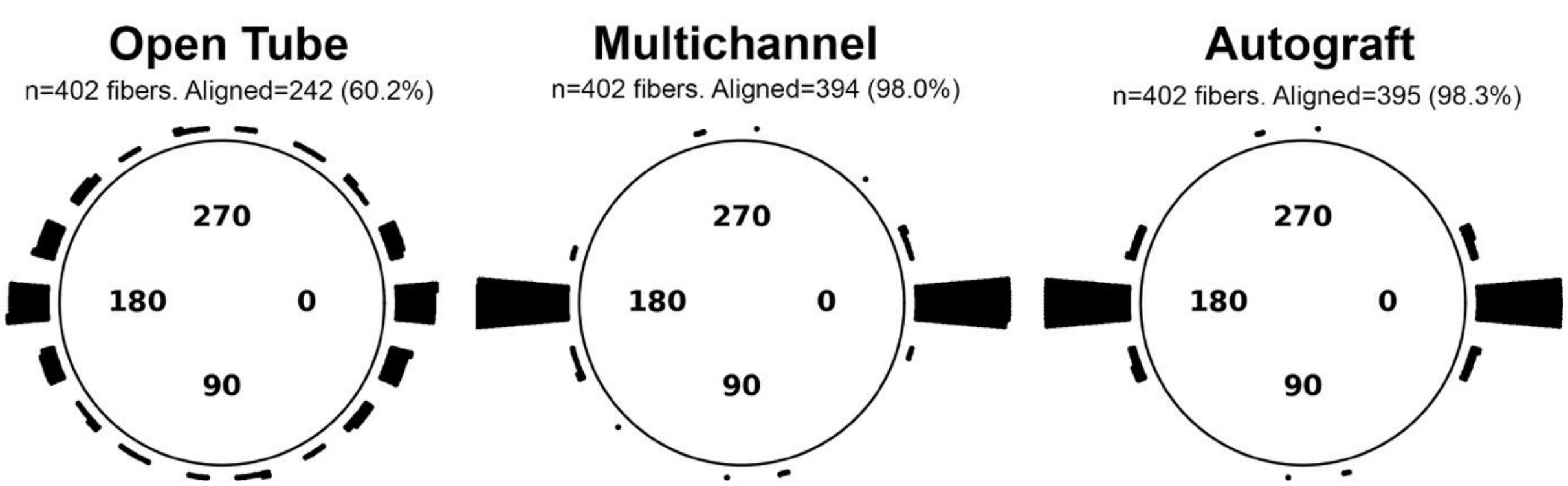

Supplemental Figure 1: Schwann cell organization following peripheral nerve repair. Circular histograms showing Schwann-cell orientation in the open tube, multichannel and autograft groups. Cellular orientation was measured relative to the longitudinal regeneration axis (proximal to distal; left to right in S100 labeled images). Dots represent individual cells grouped into 20° bins. The inner circle indicates the reference orientation axis (0°, 90°, 180°, and 270°). A total of 402 cells were analyzed per group. The proportion of aligned fibers (<20° from the regeneration axis) differed significantly among groups ($p < 0.0001$). Pairwise comparisons demonstrated significantly greater alignment in the autograft and multichannel groups compared with

open tube repair (adjusted $p < 0.0001$ for both comparisons), whereas no significant difference was detected between autograft and multichannel repair (adjusted $p = 1.0$).

2. Quantitative assessment of the immune response.

Response: Host immune response in the nerve proximal and distal to implants was evaluated using CD45 immunolabeling (detects leukocyte subclasses including T and B cells, and granulocytes) 4 weeks post sciatic nerve injury in rats (**Supp. Fig. 2)**. CD45-labeled cells were present in both proximal and distal nerve segments adjacent to the implants, and all repair strategies exhibited comparable densities of CD45 immunoreactivity, indicating a similar host immune response across treatments. Previous studies have demonstrated that peripheral nerve injury initiates a coordinated immune response during which infiltrating immune system cells contribute to axonal and myelin debris clearance, Schwann cell activation, extracellular matrix remodeling, and support of axonal regeneration[1-3]. This immune response is essential to clear the nerve and injury site of debris so that successful regeneration can occur[1,2,4]. Accordingly, the comparability among groups is expected.

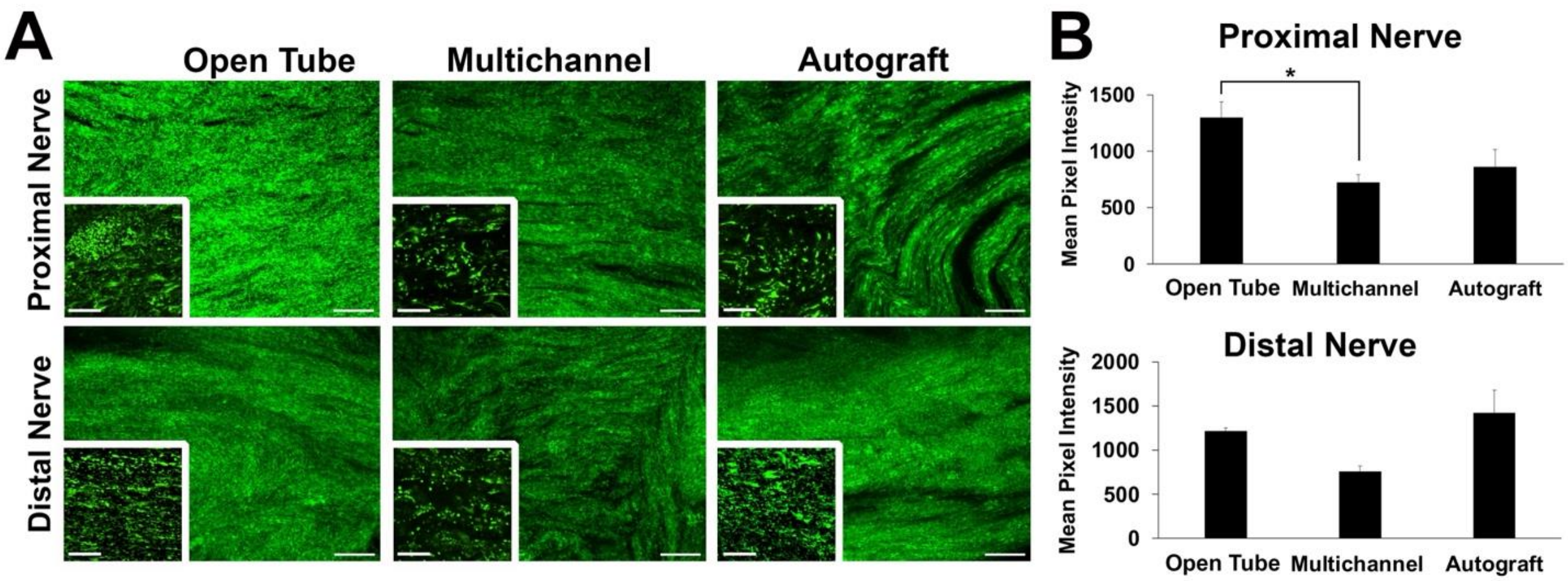

Supplemental Figure 2: Immune response in nerve tissue surrounding the implants, 4 weeks post sciatic nerve injury in rats. A. Representative longitudinal sections immunolabeled for CD45 (green) in proximal and distal nerve segments following repair with an open tube, multichannel device or autograft. Insets show higher magnification. Scalebar: 100µm and 10 µm (insets). **B.** Quantification of CD45 mean fluorescence intensity. Similar levels of CD45 immunoreactivity were observed among groups overall, in both distal and proximal nerve tissue, although a modest increase in proximal CD45 fluorescence intensity was detected in the Open Tube group compared to the Multichannel group ($p<0.04$, one-way ANOVA followed by Tukey's post hoc test). Thus, the multichannel response does not activate an immune response differing from the other treatment conditions.

3. Neuromuscular junction quantification.
Response: We quantified the number of neuromuscular junctions (NMJs) in longitudinal 35µm sections of the gastrocnemius muscle (new **Supp. Fig. 4**). We detected 19.3 ±1.7 NMJs per section in animals implanted with Open Tubes, 19.1 ± 1.7 NMJs per section in

animals implanted with Multichannel scaffolds, and 17.5 ± 2.1 NMJs per section in animals implanted with autografts. There were no statistical differences between the groups (ANOVA, p=0.8). This is expected, as studies report that NMJs remain structurally detectable for weeks and even months following denervation, and axonal reinnervation and functional recovery may not correlate with NMJ density[4,5]. Thus, quantification of NMJs does not reliably reflect degeneration and regeneration of NMJs because their presence does not guarantee restored function. Indeed, human data reported that NMJs can persist long after nerve injury, even for years [6,7]. In these patients, endplates remained visible well beyond six months, including cases lacking functional recovery[6,7]. Moreover, Sakuma et al.[5] reported that regenerating axons reach NMJs after prolonged denervation, yet functional recovery may still be absent. The lack of functional recovery in the Sakuma study was not due to failure of axonal regeneration. Thus, they concluded that even demonstrating anatomical axon–endplate contact is not a reliable quantitative endpoint for functional regeneration. It was for this reason that we did not initially perform this analysis, but in response to requests from the reviewer and editor we now provide these data that can be included in the revised manuscript as Suppl Fig 4 (below). We believe that these results are best omitted from the manuscript, because the meaning of NMJ quantification is unclear, but will include them if requested by the reviewer and editor.

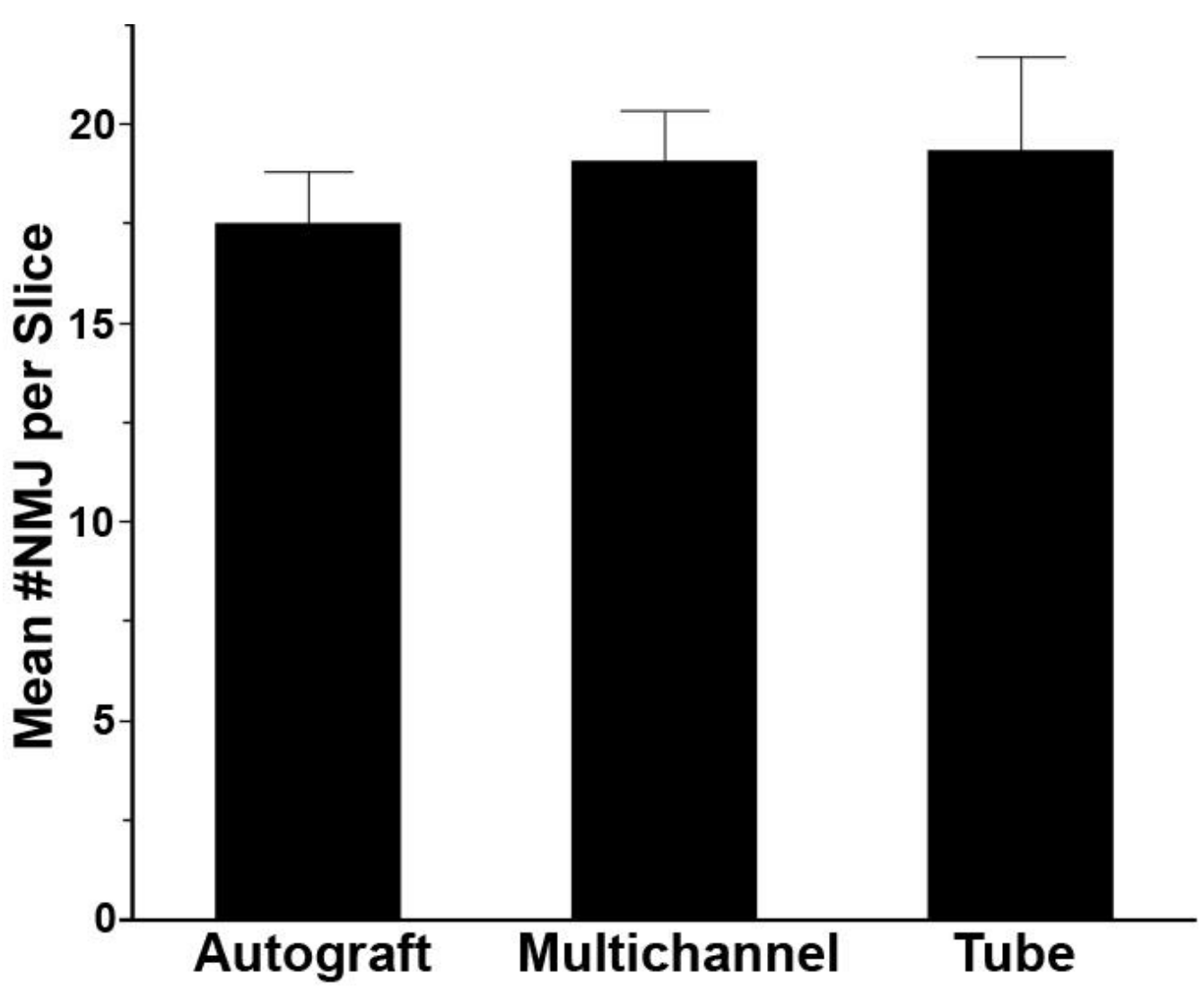

Supplemental Figure 4: Quantification of the number of neuromuscular junctions 6 months after sciatic nerve repair in rats. Quantification of α-bungarotoxin-positive neuromuscular junctions (NMJs) in gastrocnemius muscle sections following sciatic nerve injury and repair with autograft, multichannel scaffold, or tube repair. Data are presented as mean ± SEM NMJs per section. No significant differences were observed among groups (one-way ANOVA, p = 0.78).

It remains the case that electrophysiological measures, such as the compound muscle action potential (CMAP) represent a more biologically meaningful assessment because they directly evaluate whether regenerated motor axons can activate target muscles. For this reason, we believe electrophysiology provides a more relevant and interpretable outcome measure for regeneration than counting NMJs. Our electrophysiological data showed that rabbits treated with our multichannel scaffolds exhibited significantly greater muscle activation compared to open tube- and autograft-treated subjects (**Figure 8D-E**).

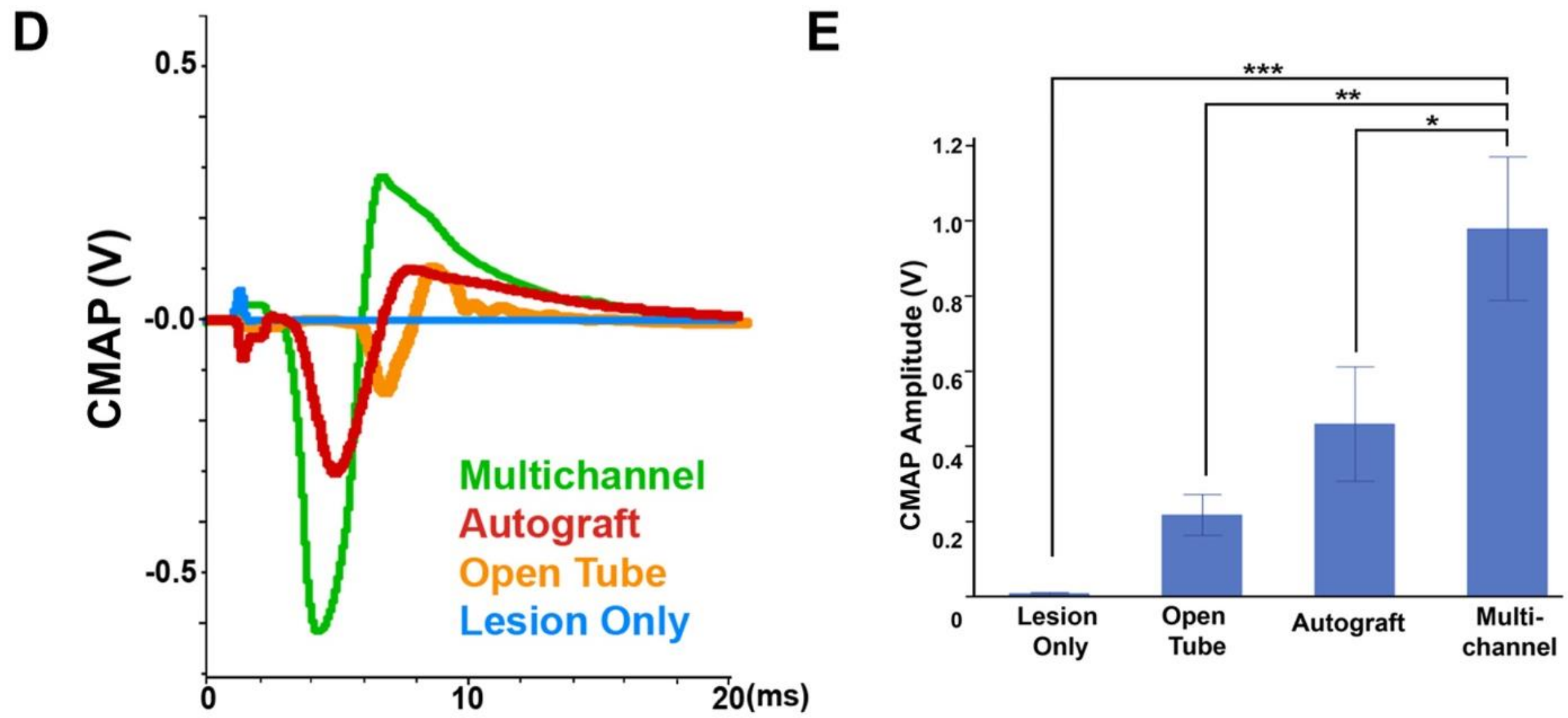

Figure 8: Rabbit studies. D-E. Animals treated with multichannel scaffolds recovered higher amplitudes of CMAPs compared to all other treatments. ANOVA P<0.0005; post-hoc Tukey's P<0.05 compared to autograft, P<0.01 compared to open tube, and P<0.001 compared to lesion only. N=11 animals/group.

Once again, we are grateful to the reviewers for their insightful comments and thank you for considering these responses.

References

1. Gaudet, A.D., Popovich, P.G. & Ramer, M.S. Wallerian degeneration: gaining perspective on inflammatory events after peripheral nerve injury. *J Neuroinflammation* **8**, 110 (2011).
2. Rotshenker, S. Wallerian degeneration: the innate-immune response to traumatic nerve injury. *J Neuroinflammation* **8**, 109 (2011).
3. Zigmond, R.E. & Echevarria, F.D. Macrophage biology in the peripheral nervous system after injury. *Prog Neurobiol* **173**, 102-121 (2019).
4. Kalinski, A.L.*, et al.* Analysis of the immune response to sciatic nerve injury identifies efferocytosis as a key mechanism of nerve debridement. *Elife* **9**(2020).
5. Sakuma, M.*, et al.* Lack of motor recovery after prolonged denervation of the neuromuscular junction is not due to regenerative failure. *Eur J Neurosci* **43**, 451-462 (2016).
6. Gupta, R.*, et al.* Human motor endplate remodeling after traumatic nerve injury. *J Neurosurg* **135**, 220-227 (2021).
7. Overland, J.*, et al.* The Fate of the Neuromuscular Junction in Peripheral Nerve Injury – Current Understanding and Future Research Aims: A Scoping Review. *JPRAS Open* (2026).

We thank the reviewer for his/her comments and provide the following responses:

1. Supp Figure 1: it is unclear how many independent biological replicates (animals) were used for the analysis: the authors state "A total of 402 cells were analyzed per group", however, statistical analysis shall be performed between experimental groups considering biological replicates (each one with an X number of cells that were counted). These details need to be provided.
I cannot see representative images reflecting the graphs.

Response: The analysis was performed using **N = 6 animals per treatment group**.

Multiple images were acquired from each animal, and an equal number of Schwann cells were randomly sampled from each image using the same predefined analysis pipeline. To avoid regional sampling bias, equal numbers of cells were selected from the top, middle, and bottom thirds of each image, and the total number of quantified cells was subsequently equalized across treatment groups. Consequently, a total of 402 cells were included in the final analysis for each treatment group.

Our analysis of individual cell orientations follows an approach that has been used previously for orientation analyses in the literature, where large numbers of individual neurites or cells are quantified to characterize orientation distributions using circular histograms and circular statistical methods. For example, Lu et al. quantified the orientation of individual neurites to characterize directional organization after spinal cord injury (Lu et al., *Stem Cell Reports*, 2019)[1].

Representative images of Schwan cell alignment are in figure 3.

2. Supp Figure 2: the authors used a general immune cell marker, CD45, which does not allow drawing biologically significant conclusions since all immune cells are CD45 positive. It is also not clear how many biological replicates were used and if the statistical analysis was based on independent biological replicates.

Response: The analysis was performed using **N = 9 animals per treatment group**.

In the original review, we were asked to "characterize the immune response" to the implanted materials. The objective of this analysis was to determine whether the Multichannel device elicited an increased overall host immune response compared with the control repair strategies, rather than to perform immune-cell phenotyping. CD45 is a well-established pan-leukocyte marker that labels infiltrating leukocytes and is widely used to quantify total immune-cell recruitment following tissue injury and repair. Accordingly, CD45 provides an accepted measure of the overall host immune response, which was the specific endpoint evaluated in the present study. Comprehensive studies of the immune response following peripheral nerve injury similarly use CD45 to define

the total leukocyte population as demonstrated by Kalinski et al. (eLife, 2020)[2]. This approach is also consistent with the FDA-recognized ISO 10993 standards used for the biological evaluation of implantable medical devices.

3. Supp Figure 4: as above: It is not clear how many biological replicates were used and if the statistical analysis was based on independent biological replicates.

Response: The analysis was performed using **N = 11 animals per treatment group**.

References

1. Lu, P., *et al.* Origins of Neural Progenitor Cell-Derived Axons Projecting Caudally after Spinal Cord Injury. *Stem Cell Reports* **13**, 105-114 (2019).
2. Kalinski, A.L., *et al.* Analysis of the immune response to sciatic nerve injury identifies efferocytosis as a key mechanism of nerve debridement. *Elife* **9**(2020).
